# Supplementary material for: Click-to-Release for Controlled Immune Cell Activation: Tumor-Targeted Unmasking of an IL12 Prodrug
Source: Pharmaceuticals (Basel). 2025 Sep 16;18(9):1380. doi: 10.3390/ph18091380 (PMC12472795; doi:10.3390/ph18091380)
Supplement: Supplementary file 1 [file pharmaceuticals-18-01380-s001.zip › pharmaceuticals-3805706-supplementary.pdf]

pharmaceuticals

Supplementary Information

Click-to-Release for Controlled Immune Cell Activation: Tumor-Targeted Unmasking of an IL12 Prodrug

Table of Contents	page
Methods	2
Organic Synthesis	6
Supplementary Figures	17
Spectra	20

Methods

General

All reagents, chemicals, materials, and solvents were obtained from commercial sources and were used as received, including nitrile starting compounds that have not been described. All solvents were of AR quality. IL12 (native human sequence produced in HEK cells) was obtained from PeproTech (Cranbury, NJ, USA) or WuXi Biologics (Jiangsu, China). [^{111}In]Indium chloride and sodium [^{125}I]iodide solutions were purchased from Curium (Petten, The Netherlands) and Revvity (Groningen, the Netherlands), respectively. Sulfo-Cy5-TCO was purchased from Broadpharm (San Diego, CA, USA). PEG reagents were purchased at Broadpharm, Creative Pegworks (Chapel Hill, NC, USA), and NOF EUROPE (Frankfurt am Main, Germany). Compound **2** (TCO-4-PEG$_2$-maleimide) was purchased from SiChem (Bremen, Germany). Compound **3** (TCO-oxymethylacetamide or TCO-Ac) was prepared as described in Rossin et al. [43]. Compound **S2** (*t*-butyl (4-(6-(1-(((4-nitrophenoxy)carbonyl)oxy)ethyl)-1,2,4,5-tetrazin-3-yl)benzyl)carbamate) was prepared as described in [27]. Compound **S6** was prepared as described in patent WO2020256546. Compound **S9** (6-(6-(pyridin-2-yl)-1,2-dihydro-1,2,4,5-tetrazin-3-yl)pyridin-3-amine) was prepared as described in [44]. Amicon Ultra centrifugal devices (MW cut-off 10 and 30 kDa) were purchased from Millipore (Burlington, MA, USA).

Analytical thin-layer chromatography was performed on Kieselgel F-254 precoated silica plates from Sigma Aldrich (Saint Louis, MO, USA). Column chromatography was carried out on Screening Devices B.V. silica gel (flash: 40–63 μm mesh; normal: 60–200 μm mesh (Amersfoort, the Netherlands)). ^{1}H NMR and ^{13}C NMR spectra were recorded on a Bruker Avance III HD spectrometer (400 MHz for ^{1}H NMR and 100 MHz for ^{13}C NMR, (Bruker, Billerica, MA, USA)) or a JEOL spectrometer (500 MHz for ^{1}H NMR, (JEOL, Tokyo, Japan)) at 298 K. Chemical shifts are reported in ppm downfield from TMS at rt. Abbreviations used for splitting patterns are s = singlet, d = doublet, dd = double doublet, t = triplet, q = quartet, m = multiplet, and br = broad. HPLC-PDA/MS was performed using a Shimadzu LC-10 AD VP series HPLC (Shimadzu, Kyoto, Japan) coupled to a diode array detector (Finnigan Surveyor PDA Plus detector (Thermo Electron Corporation, Waltham, MA, USA)) and an Ion-Trap (LCQ Fleet, Thermo Fisher Scientific). HPLC-analyses were performed using a Alltech Alltima HP C_{18} 3μ column (Alltech, Nicholasville, KY, USA) using an injection volume of 1–4 μL, a flow rate of 0.2 mL/min and typically a gradient (5% to 100% in 10 min, held at 100% for a further 3 min) of MeCN in H_2O (both containing 0.1% formic acid) at 298 K. Size exclusion chromatography (SEC) was performed on an Akta system equipped with a Superdex75 10/300 or a 16/600 (Hi-Load) column (Cytiva, Marlborough, MA, USA) eluted at 0.5–1.0 mL/min with PBS. Radio-HPLC was performed on an 1200 Infinity system (Agilent, Santa Clara, CA, USA), equipped with a Gabi Star radioactive detector (Elysia-Raytest, Straubenhardt, Germany). The samples were loaded on an Alltima C_{18} column (4.6 × 250mm, 5μ; HiChrom (Avantor, Radnor, PA, USA)), which was eluted at 1 mL/min with a linear gradient of water (A) and MeCN (B) containing 0.1% TFA v/v% (Gradient 1: 3 to 20% B in 3 min followed by 20 to 40% B in 26 min; Gradient 2: 3 to 20% B in 4 min followed by 20 to 70% B in 26 min; Gradient 3: 3 to 20% B in 4 min followed by 20 to 40% B in 86 min). Radio-ITLC was performed on ITLC-SG strips (Varian Inc., Palo Alto, CA, USA) eluted with 200 mM EDTA in saline solution (^{111}In-labeling) or MeOH/EtOAc 1:1 (^{125}I-labeling). The radioactivity distribution on ITLC strips was monitored with a Typhoon FLA 7000 phosphor imager (Cytiva) using the AIDA software v4.2. In 200 mM EDTA, the ^{111}In-labeled tetrazine remains at the base while unbound ^{111}In migrates with an R_f of 0.9. In MeOH/EtOAc the ^{125}I-labeled proteins remain at the base while

^{125}I-SHPP (Bolton-Hunter reagent, Pierce/Thermo Fisher Scientific, Waltham, MA, USA) migrates with an R_f 0.5–0.9. SDS-PAGE was performed on a Mini-PROTEAN Tetra Cell system using 4–20% precast Mini-PROTEAN TGX gels and Precision Plus Protein All Blue protein standards (BioRad Laboratories, Hercules, CA, USA). The gels were stained with Coomassie Brilliant Blue for protein detection.

Experiments involving animals

The animal study protocol was approved by the institutional Animal Welfare Committee of the RadboudUMC (project number 2019-0037, approved on 21 December 2020). Female mice (BALB/cAnNRj-Foxn1nu/Foxn1nu, Janvier Laboratories) were used for the experiments. Upon arrival, the animals were randomly allocated to polysulfone IVC cages (up to 6 mice per cage) with environmental enrichment (autoclaved tissue paper and clubhouse) and provided with standard rodent chow and water ad libitum. The cages were housed at 20–26°C and 40–70% humidity with a 12 h light/dark cycle. All animals were uniquely identified with a tattoo. After 1 week of acclimatization, the mice were inoculated s.c. on the right hindlimb with LS174T tumor cell suspension. When the tumors were 0.25–0.30 cm^3 size (ca. 2 weeks after inoculation), the animals were randomly allocated to the experimental groups using a randomized block design. The treated animals were kept in the original cage, and all cages were stored on adjacent shelves of the IVC rack. The animals were checked daily for any effects of tumor growth and treatments on behavior such as mobility, food and water consumption, and any other abnormalities. Mortality and observed clinical signs for individual animals were recorded. All animal procedures and measurements were performed by blinded biotechnicians.

Animals were euthanized if they reached one of the following humane endpoints:

- >20% body weight loss relative to the weight at the beginning of the study or >15% body weight loss in two consecutive measurements;
- tumor ulcerations causing discomfort;
- general animal poor health (emaciation, labored breathing, pale skin, hindlimb paralysis or weakness, etc.).

Sample sizes were determined a priori and were based on the minimal number of animals needed to provide a basic view of the concept.

IL12 conjugation with Tz-PEG masking moieties

A stock solution of hIL12 was added with 20 molar equivalents of the PFP-activated Tz-PEG masking compound **1** (10 mM in dry DMSO) and diluted with PBS to a 2 mg/mL final protein concentration. The pH was adjusted to ca. 8.5 with 0.1M sodium carbonate and the reaction mixture was incubated overnight at rt in the dark under gentle shaking. The IL12-Tz-PEG conjugate **4** was then purified by SEC. The collected fractions were concentrated using Amicon centrifugal devices (MW cut-off 30 kDa, Sigma Aldrich) and the conjugate concentration in the obtained solution was measured using a BCA assay.

Functionalization grade measurement of IL12-Tz-PEG 4

1 μg aliquots of the IL12-Tz-PEG conjugates were reacted with a known excess of sulfo-Cy5-TCO (Broadpharm) in 30 μL PBS. After 2 h incubation at 37°C, the mixture was analyzed by SEC with absorbance measurement at 600 nm. The IL-12 functionalization grade (FG) was then determined from the ratio between the free sulfo-Cy5-TCO peak and that of the reaction products in the chromatogram. The addition of 20 molar equivalents of **1** led to an IL12 functionalization grade of 10.6.

Anti-TAG72 diabody functionalization with TCO moieties

The anti-TAG72 diabody (AVP0458) was functionalized with TCO-PEG$_2$-maleimide (compound **2**) as previously described [28]. Briefly, the diabody was reduced with 6 mM DTT for 2 h at rt followed by purification via a PD-10 column, pre-equilibrated with 100 mM phosphate buffer pH 6.8, containing 2 mM EDTA. The reduced diabody with 4 reactive cysteines was then added with TCO-PEG$_2$-maleimide **2** (7.5 eq. per SH) dissolved in dry DMSO. The mixture was incubated for 3 h at rt. The TCO-functionalized diabody was then purified via a PD-10 column, pre-equilibrated with PBS. The obtained solutions of conjugate **5** were concentrated via Amicon centrifugal devices and a BCA assay was used to measure the protein concentration. HPLC-QTOF-MS analysis confirmed the presence of four TCO moieties per diabody molecule (Figure. S2).

IL12 conjugation with TCO-PEG masking moieties

A stock solution of IL12 in PBS was added with various amounts of the PFP-activated TCO-PEG masking moieties (in dry DMSO; **6** and **7**: 10 mg/mL; see the table in Figure 2c for equivalents added) and diluted with PBS to a 0.40 mg/mL final protein concentration. The pH was adjusted to ca 8.5 with sodium carbonate and the reaction mixtures were incubated for 1–2 h at rt in the dark under gentle shaking followed by overnight incubation at 4°C. The IL12-TCO-PEG conjugates were then purified by SEC. The collected fractions were concentrated using Amicon centrifugal devices (MW cut-off 30 kDa) and the concentration of conjugates **10** and **11** in the obtained solutions was measured using a BCA assay (Thermo Fisher Scientific).

Functionalization grade measurement of IL12-TCO-PEG 10 and 11

The functionalization grades (FG) of IL12-TCO-PEG conjugates were measured by a tetrazine titration method [45]. with minor modifications.

(2,2′,2′′-(10-(2,40,44-Trioxo-44-((6-(6-(pyridine-2-yl)-1,2,4,5-tetrazin-3-yl)pyridine-3-yl)amino)-6,9,12,15,18,21,24,27,30,33,36-undecaoxa-3,39-diazatetratetracontyl)-1,4,7,10-tetraazacyclododecane-1,4,7-triyl)triacetic acid) was prepared as described (DOTA-Tz). 2 µg aliquots of the IL12-TCO-PEG conjugates were reacted with a known excess of ^{111}In-labeled DOTA-Tz in 40 µL PBS. After 2 h incubation at 37°C, the proteins were precipitated from the reaction mixture by adding 50 µL ice-cold MeCN. After 30 min at −20°C, the vials were centrifuged at 14000 rpm, the supernatant was two-fold diluted with H_2O, and analyzed by RP-HPLC using gradient 1 (IL12-TCO-PEG$_2$ **10**) or gradient 2 (IL12-TCO-PEG$_{1K}$ **11**). The IL12 functionalization grades were then determined from the ratio between the free ^{111}In-tetrazine peak and that of the released reaction product in the radiochromatogram.

Anti-TAG72 diabody functionalization with tetrazine moieties

The anti-TAG72 diabody (AVP0458) was functionalized with tetrazine-PEG-maleimide (**9**) as previously described for other maleimides [28]. Briefly, the diabody was reduced with 6 mM DTT for 2h at rt followed by purification via a PD-10 column, pre-equilibrated with 100 mM phosphate buffer pH 6.8, containing 2 mM EDTA. The reduced diabody with 4 reactive cysteines was then added with tetrazine **9** (5 eq. per SH) dissolved in dry DMSO. The mixture was incubated overnight at 4°C. The Tz-functionalized diabody **12** was then purified by SEC. The collected fractions were concentrated via Amicon centrifugal devices and a BCA assay was used to measure the protein concentration. HPLC-QTOF-MS analysis confirmed the presence of four Tz moieties per diabody molecule (Figure. S2).

Data analysis

Statistical analysis on IL12 ELISA data was performed using two-way ANOVA using GraphPad Prism software version 10.5.0 for Windows. Significance is indicated by ns ($p > 0.05$), * ($p \leq 0.05$), ** ($p \leq 0.01$), *** ($p \leq 0.001$).

Organic Syntheses

1-{6-[4-({[(Tert-butoxy)carbonyl]amino}methyl)phenyl]-1,2,4,5-tetrazin-3-yl}ethyl N-[4-(hydroxymethyl)phenyl]-N-methylcarbamate (S3)

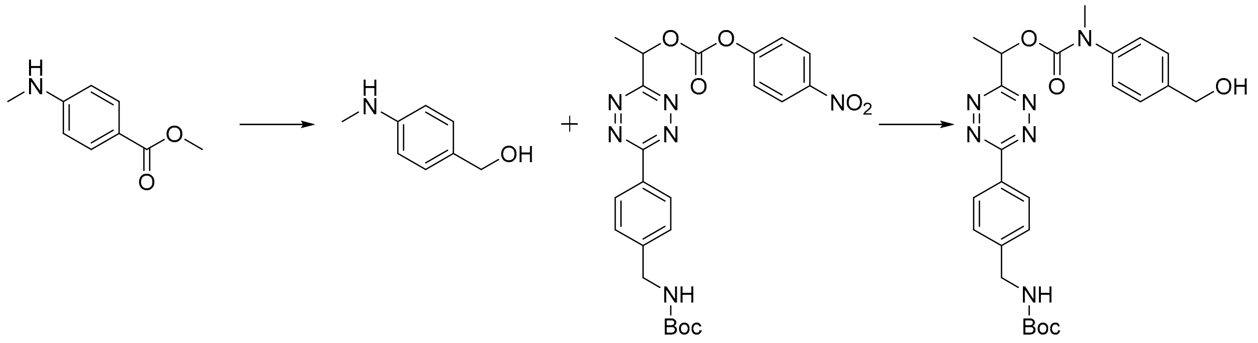

S1 **S2** **S3**

Methyl 4-(methylamino)benzoate (82.6 mg, 500 µmol) was dissolved in dry THF (2.5 mL). $LiAlH_4$ (38.6 mg, 1.02 mmol) was added and the mixture was stirred overnight at rt. The mixture was cooled to 0°C and quenched by the addition of water (5 mL). The reaction was stirred for 40 min, filtered, and the filtrate was concentrated in vacuo. The residue was redissolved in CH_2Cl_2 (30 mL), dried over $MgSO_4$, and filtered. Following concentration, the reaction mixture was applied to a silica column in CH_2Cl_2 and eluted over a CH_2Cl_2:EtOAc gradient (100:0 to 80:20) to afford the intermediate **S1** as a yellow oil (38.3 mg, 279 µmol, 55.8%). **S1** (35 mg, 0.26 mmol) was dissolved in CH_2Cl_2/pyridine (9:1 v/v, 1.5 mL). Chlorotrimethylsilane (90 µL, 0.71 mmol) was added and the mixture was stirred for 1h at rt. Subsequently, compound **S2** (24 mg, 48 µmol), 1-hydroxybenzotriazole hydrate (2 mg, 0.01 mmol), and DiPEA (90 µL, 0.52 mmol) were added and the mixture was stirred overnight at rt. MeOH/H_2O (1:1 v/v, 4 mL) was added and the mixture was stirred for 30 min at rt. The solvent was removed in vacuo, followed by redissolving in CH_2Cl_2 (20 mL). The organic phase was washed with water (10 mL), 0.1 M HCl (3 × 10 mL), sat. $NaHCO_3$ (5 × 10 mL) and brine (10 mL), dried over Na_2SO_4 and filtered. Following concentration, the reaction mixture was applied to a silica column in CH_2Cl_2 and eluted over a CH_2Cl_2:EtOAc gradient (100:0 to 75:25) to afford **S3** as a pink solid (19.3 mg, 39.0 µmol, 81%). Analysis **S1**: ^1H NMR (500 MHz, $CDCl_3$) δ 7.19 (d, J = 8.5 Hz, 2H), 6.60 (d, J = 8.5 Hz, 2H), 4.53 (s, 2H), 2.83 (s, 3H) ppm. Analysis **S3**: ^1H NMR (500 MHz, $CDCl_3$) δ 8.56 (d, J = 8.5 Hz, 2H), 7.49 (d, J = 8.0 Hz, 2H), 7.34 (s, 4H), 6.25 (q, J = 7.1 Hz, 1H), 5.04 (s, 1H), 4.67 (s, 2H), 4.43 (d, J = 6.1 Hz, 2H), 3.34 (s, 3H), 1.79 (s, 3H), 1.48 (s, 9H) ppm.

1-{6-[4-(Aminomethyl)phenyl]-1,2,4,5-tetrazin-3-yl}ethyl N-[4-(hydroxymethyl)phenyl]-N-methylcarbamate (S4)

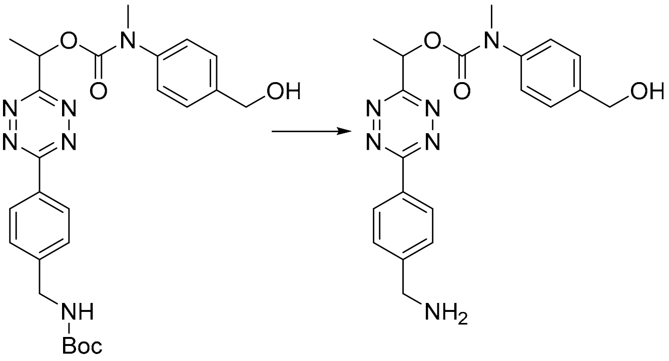

S3 **S4**

Compound **S3** (19 mg, 38 µmol) was dissolved in CH_2Cl_2 (1.5 mL). TFA (0.5 mL) was added and the mixture was stirred for 1h at rt. Following concentration, the reaction mixture was applied to a silica column in CH_2Cl_2 and eluted over a CH_2Cl_2:MeOH gradient (100:0 to 80:20) to afford the TFA salt of **S4** as a pink solid (9.0 mg, 18 µmol, 46%). ^{1}H NMR (500 MHz, CD_3OD): δ 8.74–8.63 (m, 2H), 7.79–7.71 (m, 2H), 7.39 (d, *J* = 14.7 Hz, 4H), 6.20 (d, *J* = 12.2 Hz, 1H), 4.62 (d, *J* = 18.4 Hz, 2H), 4.29 (d, *J* = 18.5 Hz, 2H), 3.34 (s, 3H), 1.76 (s, 3H) ppm.

1-(6-{4-[(2,5,8,11,14,17,20,23,26,29-Decaoxadotriacontan-32-amido)methyl]phenyl}-1,2,4,5-tetrazin-3-yl)ethyl N-[4-(hydroxymethyl)phenyl]-N-methylcarbamate (S5)

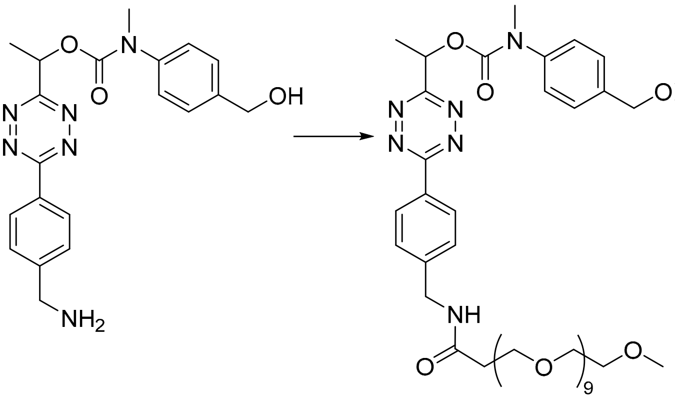

S4 **S5**

Compound **S4** (9.0 mg, 18 µmol) and mPEG$_{10}$-COOH (21.5 mg, 42.9 µmol) were dissolved in dry DMF (2 mL) and DiPEA (30 µL, 0.17 mmol). PyBOP (52 mg, 0.10 mmol) was added and the mixture was stirred overnight at rt. CH_2Cl_2 (15 mL) was added and the organic phase was washed with 0.1 M HCl (3 × 10 mL), sat. $NaHCO_3$ (3 × 10 mL) and brine (10 mL), dried over Na_2SO_4 and filtered. Following concentration, the reaction mixture was applied to a silica column in CH_2Cl_2 and eluted over a CH_2Cl_2:MeOH gradient (100:0 to 90:10) to afford **S5** as a pink solid (8.4 mg, 90% purity, 8.6 µmol, 49%). ^{1}H NMR (500 MHz, $CDCl_3$): δ 8.54 (d, *J* = 6.9 Hz, 2H), 7.52 (d, *J* = 7.4 Hz, 2H), 7.34 (d, *J* = 3.7 Hz, 4H), 6.25 (q, *J* = 6.8 Hz, 1H), 4.67 (s, 2H), 4.58 (d, *J* = 5.4 Hz, 2H), 3.79 (t, *J* = 5.2 Hz, 2H), 3.67–3.51 (m, ca. 51H), 3.37 (dd, *J* = 4.2, 0.8 Hz, 6H), 2.63–2.57 (m, 2H), 1.80 (s, 3H) ppm.

[4-({[1-(6-{4-[(2,5,8,11,14,17,20,23,26,29-Decaoxadotriacontan-32-amido)methyl]phenyl}-1,2,4,5-tetrazin-3-yl)ethoxy]carbonyl}(methyl)amino)phenyl]methyl 2,3,4,5,6-pentafluorophenyl carbonate (1)

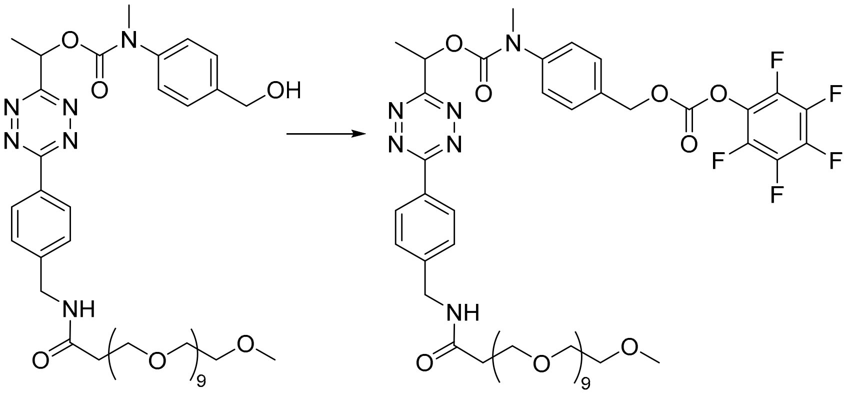

Compound **S5** (8.2 mg, 90% purity, 8.4 μmol) was dissolved in dry CH_2Cl_2 (1.5 mL). DMAP (5.1 mg, 42 μmol) and pentafluorophenyl carbonate (17.7 mg, 44.9 μmol) were added and the mixture was stirred for 1h at rt. Pentafluorophenyl carbonate (13.8 mg, 35.0 μmol) was added and the mixture was stirred for 1h at rt. CH_2Cl_2 (15 mL) was added and the organic phase was washed with 0.1 M HCl (3 × 10 mL), sat. $NaHCO_3$ (3 × 10 mL) and brine (10 mL), dried over Na_2SO_4 and filtered. Following concentration, the reaction mixture was applied to a silica column in CH_2Cl_2 and eluted over a CH_2Cl_2:MeOH gradient (100:0 to 90:10) to afford compound **1** as a pink solid (6.4 mg, 5.9 μmol, 70%). ESI-MS calculated for $C_{49}H_{63}F_5N_6O_{16}$: 1086.42; found $[M + H]^+$ 1087.32. ^1H NMR (500 MHz, $CDCl_3$): δ 8.56 (d, *J* = 8.4 Hz, 2H), 7.69 (s, 1H), 7.53 (d, *J* = 8.5 Hz, 2H), 7.42 (s, 4H), 6.27 (q, *J* = 6.8 Hz, 1H), 5.31 (s, 2H), 4.59 (d, *J* = 5.4 Hz, 2H), 3.81 (t, *J* = 5.6 Hz, 2H), 3.67–3.53 (m, ca. 39H), 3.37 (s, 6H), 2.65 (t, *J* = 5.6 Hz, 2H), 1.82 (s, 3H) ppm.

(1R,2E,6S)-6-Hydroxy-6-{[2-(2-methoxyethoxy)ethyl]carbamoyl}cyclooct-2-en-1-yl-2,3,4,5,6-pentafluorophenyl carbonate (6)

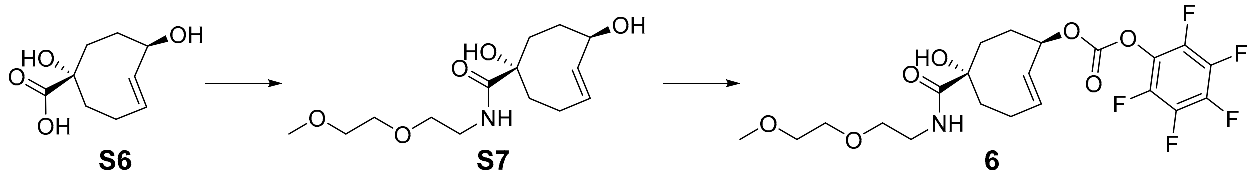

Compound **S6** (106 mg, 0.57 mmol), mPEG2-amine (136 mg, 1.14 mmol), PyBOP (296 mg, 0.57 mmol), and DiPEA (298 μL, 1.71 mmol) were dissolved in dry MeCN and stirred for 16h. Following concentration, the reaction mixture was applied to a silica column in CH_2Cl_2 and eluted over a CH_2Cl_2:MeOH gradient (100:0 to 96:4). Following concentration, the residue was redissolved in CH_2Cl_2 and dried over $MgSO_4$. Intermediate **S7** was isolated after filtration and concentration in 135.2 mg yield (0.47 mmol). **S7** (68 mg, 0.24 mmol), pentafluorophenyl carbonate (274 mg, 0.70 mmol), DMAP (1.5 mg, 12 μmol), and DiPEA (152 μL, 0.87 mmol) were dissolved in dry MeCN (2 mL) and stirred for 2 days at rt. The reaction mixture was concentrated and applied to a C_{18} reverse phase preparative HPLC column, followed by elution using a 5% to 95% gradient of MeCN in H_2O over 40 min, yielding compound **6** in 58 mg yield (0.12 mmol) after lyophilization. Analysis **S7**: ESI-MS calculated for $C_{14}H_{25}NO_5$: 287.17; found $[M + H]^+$ 288.00. ^1H NMR (400 MHz, CD_3OD): δ 6.04-5.97 (m, 1H), 5.74 (dd, *J* = 16.6, 2.5 Hz, 1H), 4.41 (m, 1H), 3.63-3.61 (m, 2H), 3.56-3.53 (m, 4H), 3.39-3.35 (m, 4H), 2.50-2.40 (m, 1H), 2.23-2.03 (m, 4H), 1.93-1.89 (m, 1H), 1.74-1.69 (m, 1H), 1.63-1.54 (m, 1H) ppm. Analysis **6**: ESI-MS calculated for $C_{21}H_{24}F_5NO_7$: 497.15; found $[M + H]^+$ 497.92, $[M + Na]^+$ 520.00. ^1H NMR (400 MHz, $CDCl_3$): δ 6.31 (s, 1H), 6.08–5.93 (m, 1H), 5.79 (dd, *J* = 16.5, 2.3 Hz, 1H), 5.34 (s, 1H), 3.63-3.61 (m, 2H), 3.58-3.53

(m, 5H), 3.49-3.45 (2H), 3.41 (s, 3H), 2.58-2.48 (m, 1H), 2.33-2.22 (m, 2H), 2.06-1.99 (m, 4H), 1.80-1.74 (m, 1H) ppm.

mPEG$_{1k}$-TCO-PFP (7)

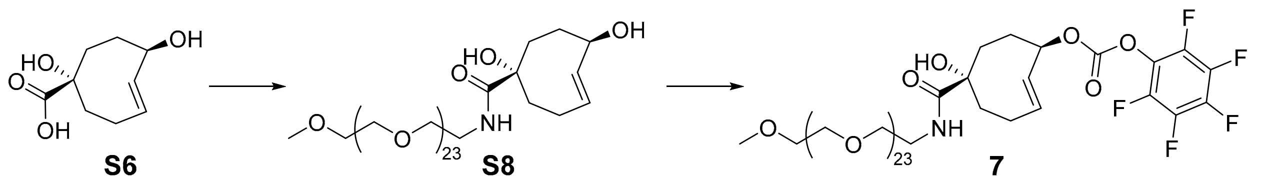

S6 S8 7

Compound **S6** (27.5 mg, 148 µmol), mPEG$_{1k}$-amine (98.1 mg, ca. 98 µmol), PyBOP (83.4 mg, 160 µmol), and DiPEA (100 µL, 574 µmol) were dissolved in dry MeCN/DMF and stirred overnight at rt. The reaction mixture was concentrated and co-evaporated with MeCN. The residue was applied to a C_{18} reverse phase preparative HPLC column followed by elution using a 5% to 95% gradient of MeCN in H_2O over 40 min. After lyophilization, the residue was dissolved in CH_2Cl_2 and dried over $MgSO_4$. Intermediate **S8** was isolated after filtration and concentration in 71 mg yield (ca. 57 µmol). **S8** (71 mg, ca. 57 µmol), pentafluorophenyl carbonate (92.1 mg, 234 µmol), DMAP (0.7 mg, 6 µmol), and DiPEA (41 µL, 0.24 mmol) were dissolved in dry MeCN and stirred for 5 days at rt. The reaction mixture was concentrated and applied to a C_{18} reverse phase preparative HPLC column, followed by elution using a 20% to 95% gradient of MeCN in H_2O over 40 min, yielding compound **7** in 7.6 mg yield (ca. 5.2 µmol) after lyophilization. Analysis **S8**: ESI-MS calculated for $C_{58}H_{113}NO_{27}$: 1255.75; found $[M + H]^+$ 1256.24. ^1H NMR (400 MHz, $CDCl_3$): δ 6.63 (s, 1H), 6.02 (ddd, *J* = 15.4, 11.3, 3.4 Hz, 1H), 5.75 (dd, *J* = 16.5, 2.3 Hz, 1H), 4.49 (s, 1H), 3.69–3.58 (m, ca. 97H), 3.55–3.51 (m, 4H), 3.36 (s, 3H), 2.46 (qd, *J* = 11.9, 4.7 Hz, 1H), 2.25 (d, *J* = 12.2 Hz, 1H), 2.12–1.89 (m, 5H), 1.74 (dd, *J* = 14.4, 6.0 Hz, 1H), 1.62 (dd, *J* = 14.9, 6.2 Hz, 1H) ppm. Analysis **7**: ESI-MS calculated for $C_{65}H_{112}F_5NO_{29}$: 1465.72; found $[M + 2H]^{2+}$ 733.64. ^1H NMR (400 MHz, $CDCl_3$): δ 6.57 (s, 1H), 6.01 (ddd, *J* = 15.4, 11.3, 3.5 Hz, 1H), 5.78 (dd, *J* = 16.6, 2.3 Hz, 1H), 5.33 (s, 1H), 3.67–3.62 (m, ca. 96H), 3.58–3.53 (m, 4H), 3.38 (s, 3H), 2.52 (qd, *J* = 12.0, 4.8 Hz, 2H), 2.35–2.21 (m, 3H), 2.14–1.94 (m, 4H), 1.78 (dd, *J* = 15.7, 5.6 Hz, 1H) ppm.

2,2′-((2-((Carboxymethyl)(2-oxo-2-((6-(6-(pyridin-2-yl)-1,2-dihydro-1,2,4,5-tetrazin-3-yl)pyridin-3-yl)amino)ethyl)amino)ethyl)azanediyl)diacetic acid (S10)

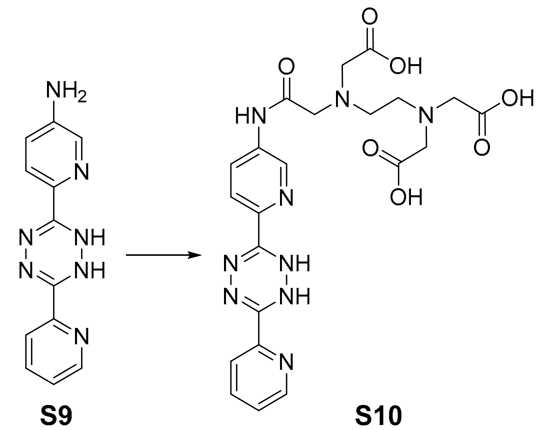

S9 S10

Ethylenediaminetetraacetic dianhydride (1.86 g, 7.26 mmol) was dissolved in dry DMSO (3 mL) by gentle heating. The mixture was allowed to cool to room temperature and a solution of **S9** (450 mg, 1.78 mmol) in DMSO (11 mL) was slowly added. The orange,

hazy mixture was stirred at room temperature under an atmosphere of argon for 5 h. Subsequently, water (0.1 mL) was added and the mixture was stirred for 30 min. **S10** mixture was used without further purification. ESI-MS: m/z Calc. for $C_{22}H_{25}N_9O_7$ 527.19 Da; Obs. $[M + H]^+$ 528.42 Da and $[M - H^+]^-$ 526.50 Da.

2,2′-((2-((Carboxymethyl)(2-oxo-2-((6-(6-(pyridin-2-yl)-1,2,4,5-tetrazin-3-yl)pyridin-3-yl)amino)ethyl)amino)ethyl)azanediyl)diacetic acid (S11)

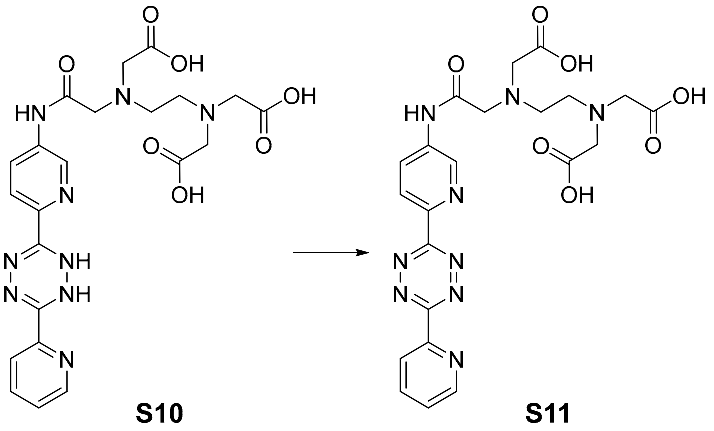

The crude reaction mixture of **S10** was diluted with water (15 mL) and acidified by the addition of formic acid (0.2 mL). Sodium nitrite (400 mg, 5.79 mmol) was added and the pink mixture was stirred in a closed flask at room temperature for 1 h. An aqueous solution of 1 M ammonium acetate (30 mL) was added and the pink suspension was centrifuged at 3000 rpm for 10 min. The clear, dark pink supernatant was isolated and purified by reversed-phase chromatography (C18 column, gradient of 5% MeCN / 0.1 M aqueous ammonium acetate to 25%). The combined product fractions were lyophilized, redissolved in water (25 mL), and again lyophilized and redissolved in water. To the pink solution was added formic acid (0.25 mL), which caused the product to precipitate. The suspension was centrifuged at 3000 rpm for 10 min, after which the clear, faint pink supernatant was discarded. The pink solid was washed with water (25 mL) and centrifuged, for two more times, and then washed with MeCN and centrifuged, for two more times. The remaining pink solid was dried in vacuo to give 486 mg (52% overall yield) of **S11**. ESI-MS: m/z Calc. for $C_{22}H_{23}N_9O_7$ 525.17 Da; Obs. $[M + H]^+$ 526.33 Da and $[M - H]^-$ 524.42 Da. ^{1}H NMR (400 MHz, DMSO-d_6): δ 12.41 (br.s, 3H), 10.78 (s, 1H), 9.13 (d, J = 2.5 Hz, 1H), 8.94 (dd, J = 4.8, 1.7 Hz, 1H), 8.62 (m, 2H), 8.52 (dd, J = 8.7, 2.5 Hz, 1H), 8.16 (td, J = 7.8, 1.8 Hz, 1H), 7.73 (dd, J = 7.8, 4.7 Hz, 1H), 3.53 (m, J = 10.4 Hz, 8H), 2.85 (s, 4H) ppm. ^{13}C NMR (101 MHz, DMSO-d6): δ 173.40, 172.97, 171.94, 163.52, 163.27, 151.08, 150.67, 144.47, 142.13, 138.49, 138.28, 127.05, 126.75, 125.28, 124.66, 58.55, 55.68, 55.37, 52.58, 52.22 ppm.

Calcium sodium 2-({2-[bis(carboxylatomethyl)amino]ethyl}[({6-[6-(pyridin-2-yl)-1,2,4,5-tetrazin-3-yl]pyridin-3-yl}carbamoyl)methyl]amino)acetate (8)

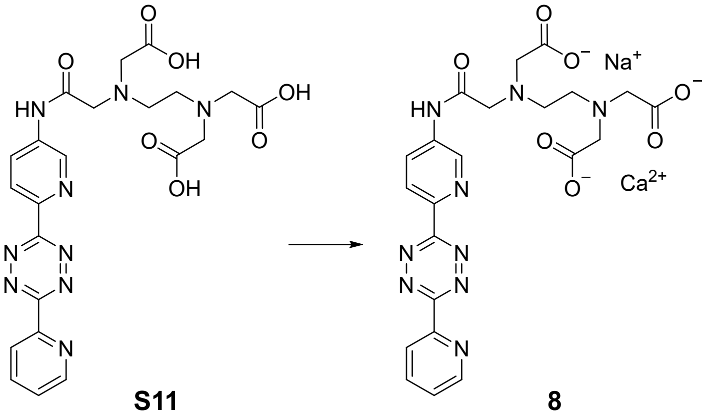

S11 **8**

1M Sodium acetate was prepared by dissolving sodium acetate trihydrate in milliQ-H_2O (pH—9.0), followed by acidification to pH = 6.4 with AcOH glacial. To an acidic suspension of **S11** (172 mg, 0.33 mmol; 10 mg/mL) in milliQ-H_2O was added dropwise $CaCO_3$ (1.67 mL, 0.53 mmol of a 32 mg/mL homogenous suspension). Upon increasing pH, the tetrazine dissolved at pH = 5.8 and $CaCO_3$ addition was halted at pH = 6.5. Subsequently, 1.0 M sodium acetate (pH = 6.4) was added to the tetrazine to obtain a final 0.1 M NaAc concentration. The solution was applied to a SEP-PAK column (10 gr, Waters) for purification (i.e., removal of excess calcium and NaAc components). The tetrazine retained at the top of the column and was rinsed once with 0.1 M sodium acetate followed by rinsing with milliQ-H_2O (6 volumes) prior to elution with milliQ-H_2O:MeOH (1:1) aided by vacuum pull. Tetrazine-containing fractions were combined, reduced 80% by volume in vacuo, diluted with milliQ-H_2O, and lyophilized after micropore filter filtration. The lyophilized residue was redissolved in milliQ-H_2O at 50 mg/mL, micropore filtered once more, and lyophilized to obtain compound **8** as a homogenous pink fluffy powder. ^1H NMR (400 MHz, D_2O) δ 8.89 (dd, *J* = 2.6, 0.6 Hz, 1H), 8.79 (ddd, *J* = 4.8, 1.7, 0.9 Hz, 1H), 8.63–8.55 (m, 2H), 8.37 (dd, *J*= 8.7, 2.6 Hz, 1H), 8.16 (td, *J* = 7.8, 1.7 Hz, 1H), 7.74 (ddd, *J* = 7.7, 4.7, 1.1 Hz, 1H), 3.58 (s, 2H), 3.32–3.06 (m, 6H), 2.77–2.50 (m, 4H) ppm. ^{13}C NMR (100 MHz, D_2O) δ 179.9, 179.3, 174.1, 162.9, 162.5, 150.3, 148.3, 143.9, 142.0, 139.0, 137.3, 128.9, 127.6, 125.3, 124.7, 60.5, 59.8, 54.8 ppm. ESI-MS cal. for $C_{22}H_{23}N_9O_7$ 525.17 (excl. sodium calcium), found. M+H$^+$ 526.25. Elemental anal. calcd for $C_{22}H_{20}CaN_9NaO_7$: Composition: C (45.1%), Ca (6.8%), N (21.5%), Na (3.9%). Measured: C (44.0%), Ca (7.2%), N (20.5%), Na (3.1%).

2-Triisopropylsilyloxymethyl-3,4-dihydro-2H-pyran (S12)

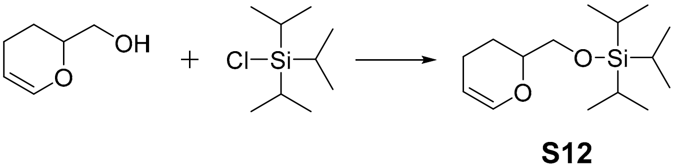

S12

3,4-Dihydro-2H-pyran-2-methanol (2.50 g, 21.9 mmol) was dissolved in DMF (30 mL). Imidazole (3.73 g, 55 mmol) was added, and the mixture was cooled to 0°C. Triisopropylsilyl chloride (4.86 g, 25.2 mmol) was added, and the mixture was stirred under an atmosphere of argon at 0°C for 15 min. Then it was allowed to warm to 20°C and stirred for 4 hrs. Water (90 mL) was added and the turbid mixture was extracted with Et_2O (2×50 mL). The organic phase was dried over $MgSO_4$, filtered, and concentrated in vacuo. The crude product was purified by distillation (140°C, 0.5 mbar), to yield the product **S12** as a colorless liquid (5.26 g, 89%). ^1H NMR (400 MHz, $CDCl_3$): δ 6.36 (d, 1H), 4.67 (m, 1H), 3.92–

3.82 (m, 2H), 3.69 (m, 1H), 2.15–2.02 (m, 1H), 2.02–1.89 (m, 2H), 1.67 (m, 1H), 1.07 (m, 21H) ppm. ^{13}C NMR (101 MHz, $CDCl_3$): δ 143.59, 100.48, 75.58, 65.69, 24.54, 19.24, 17.97, 11.97 ppm.

2-Triisopropylsilyloxymethyl-3,4-dihydro-2H-pyran-6-yltributylstannane (S13)

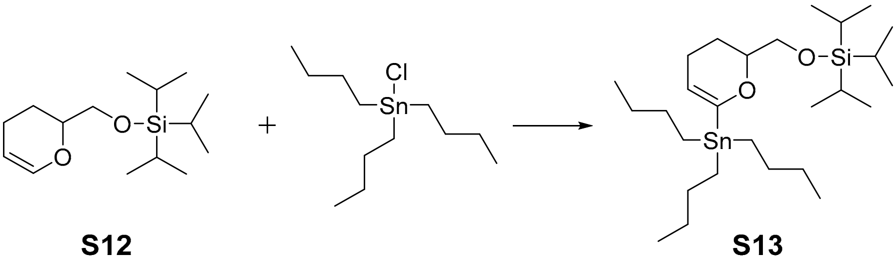

S12 **S13**

Potassium *tert*-butoxide (1.00 g, 8.92 mmol) was suspended in pentane (15 mL) and cooled to −78°C. Tetramethylethylenediamine (1.38 g, 11.88 mmol) was added, and n-BuLi (5.57 mL 1.6 M solution in hexane, 8.92 mmol) was added dropwise. After stirring for 10 min, the mixture was allowed to warm to −15°C to give a clear, yellow solution. Subsequently, it was recooled to −78°C, and **S12** (1.60 g, 5.94 mmol) dissolved in pentane (1.5 mL) was added dropwise. The mixture was allowed to warm to −15°C over 1h to give a precipitate. The turbid mixture was recooled to −78°C, and tributyltin chloride (3.86 g, 11.88 mmol) was added. The turbid, yellow mixture was allowed to warm to 20°C and quenched with saturated NH_4Cl (aq). The organic phase was isolated, dried over $MgSO_4$, and concentrated to give a residue which was purified by flash column chromatography (silica neutralized with EtN_3, hexane) to afford the product **S13** as a colorless oil (3.08 g, 93%). ^{1}H NMR (400 MHz, $CDCl_3$): δ 4.71 (m, 1H), 3.78 (m, 2H), 3.65 (m, 1H), 2.10 (m, 1H), 1.95 (m, 2H), 1.66 (m, 1H), 1.51 (m, 6H), 1.31 (m, 6H), 1.07 (m, 21H), 0.89 (m, 15H) ppm. ^{13}C NMR (101 MHz, $CDCl_3$): δ 162.10, 111.87, 75.60, 66.05, 29.00, 27.22, 24.79, 20.94, 18.00, 13.72, 12.00, 9.44 ppm.

3-(2-Triisopropylsilyloxymethyl-3,4-dihydro-2H-pyran-6-yl)-6-(3,4-dihydro-2H-pyran-6-yl)-1,2,4,5-tetrazine (S14)

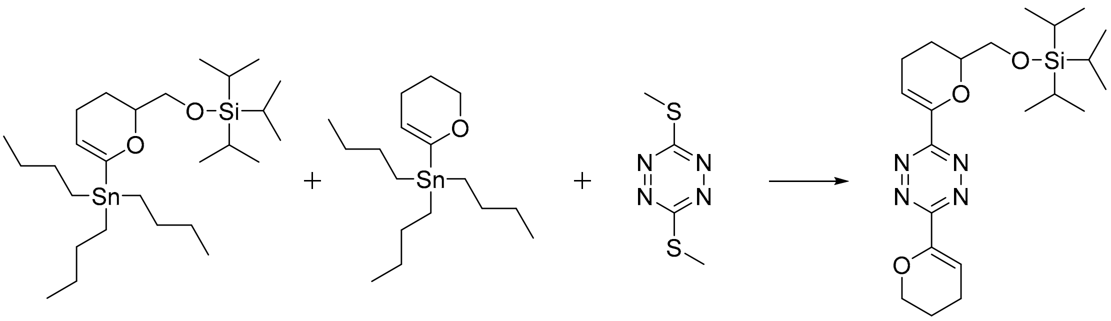

S13 **S14**

3,6-Bis-(methylthio)-1,2,4,5-tetrazine (164 mg, 0.94 mmol) was dissolved in dioxane (70 mL), and copper(I) thiophene-2-carboxylate (714 mg, 3.76 mmol), tetrakis(triphenylphosphine)-palladium(0) (163 mg, 0.14 mmol), tributyl(5,6-dihydro-4H-pyran-2-yl)stannane (700 mg, 1.88 mmol), and **S13** (1050 mg, 1.88 mmol) were added. The dark suspension was heated at 100°C under an atmosphere of argon for 90 min. After allowing to cool to 20°C, it was filtered over diatomaceous earth, concentrated in vacuo, and redissolved in CH_2Cl_2 (10 mL). The pink solution was washed with Na_2CO_3(aq) (2×15 mL), dried over Na_2SO_4, filtered, and concentrated. The crude product was purified by flash

column chromatography (silica, hexane/CH_2Cl_2) to afford **S14** as a pink oil (64 mg, 16%). ESI-MS: m/z Calc. for $C_{22}H_{36}N_4O_3Si$ 432.26 Da; Obs. $[M + H]^+$ 433.33 Da. ^{1}H NMR (400 MHz, $CDCl_3$): δ 6.70 (t, J = 4.4 Hz, 2H), 4.34 (t, J = 5.1 Hz, 2H), 4.23 (dddd, J = 9.7, 7.2, 4.5, 2.4 Hz, 1H), 4.12 (dd, J = 9.9, 4.5 Hz, 1H), 3.87 (dd, J = 9.9, 7.5 Hz, 1H), 2.41 (m, 4H), 2.22 (m, 1H), 2.04 (m, 2H), 1.88 (m, 1H), 1.18–1.00 (m, 21H) ppm. ^{13}C NMR (101 MHz, $CDCl_3$): δ 160.81, 160.62, 146.70, 146.32, 110.57, 110.46, 67.08, 64.91, 23.66, 21.82, 21.16, 20.63, 17.99, 17.97, 11.93 ppm.

3-(Hydroxymethyl-3,4-dihydro-2H-pyran-6-yl)-6-(3,4-dihydro-2H-pyran-6-yl)-1,2,4,5-tetrazine (S15)

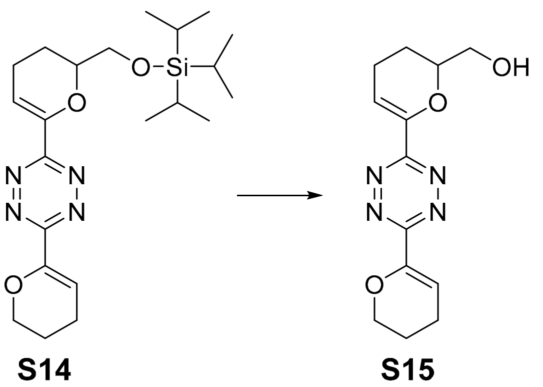

Compound **S14** (40 mg, 0.093 mmol) was dissolved in MeCN (2 mL) and diluted with water (2 mL). Formic acid (0.080 mL) was added, and the mixture was stirred at 20°C for 68 hrs. The solvent was removed in vacuo, and the residue was purified by reversed-phase column chromatography (C_{18}, water/MeCN) and lyophilization to give **S15** as a pink solid (27 mg, 100%). ESI-MS: m/z Calc. for $C_{13}H_{16}N_4O_3$ 276.12 Da; Obs. $[M + H]^+$ 277.17 Da. ^{1}H NMR (400 MHz, $CDCl_3$): δ 6.73 (m, 2H), 4.35 (dd, J = 6.2, 4.2 Hz, 2H), 4.24 (ddt, J = 9.8, 6.2, 3.0 Hz, 1H), 3.89 (qd, J = 12.0, 4.9 Hz, 2H), 2.65 (br.s, 1H), 2.58–2.35 (m, 4H), 2.04 (dt, J = 10.3, 6.1 Hz, 2H), 1.99 (m, 1H), 1.92 (m, 1H) ppm. ^{13}C NMR (101 MHz, $CDCl_3$): δ 146.64, 146,20, 110.76, 110.62, 67.10, 65.10, 23.06, 21.80, 21.18 ppm.

{6-[6-(3,4-Dihydro-2H-pyran-6-yl)-1,2,4,5-tetrazin-3-yl]-3,4-dihydro-2H-pyran-2-yl}methyl 2,5-dioxopyrrolidine-1-carboxylate (S16)

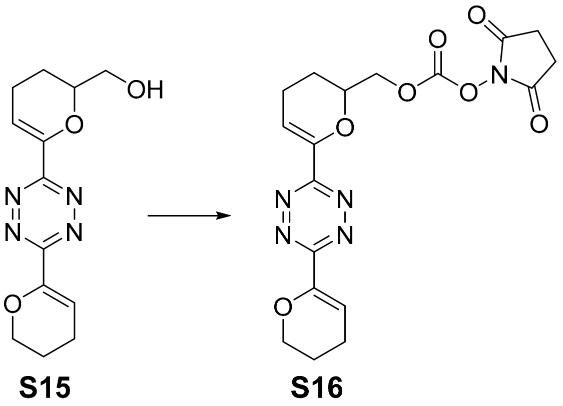

Compound **S15** (8.6 mg, 0.0312 mmol) was dissolved in MeCN (1 mL), and bis(2,5-dioxopyrrolidin-1-yl) carbonate (16 mg, 0.0624 mmol), DIPEA (12 mg, 0.094 mmol), and DMAP (0.4 mg, 0.0031 mmol) were added. The mixture was stirred at 20°C overnight, and then concentrated, redissolved in CH_2Cl_2 (2 mL), and washed with 0.5 M citric acid (aq)

(2 × 1 mL) and $NaHCO_3$ (2 × 1 mL). The pink solution was dried over Na_2SO_4, filtered, and concentrated to yield **S16** as a pink solid (13 mg, 95%). ESI-MS: m/z Calc. for $C_{18}H_{19}N_5O_7$ 417.13 Da; Obs. $[M + H]^+$ 418.25 Da. ^1H NMR (400 MHz, $CDCl_3$): δ 6.72 (m, 2H), 4.62 (m, 2H), 4.46 (m, 1H), 4.35 (dd, J = 6.2, 4.2 Hz, 2H), 2.85 (s, 4H), 2.49 (m, 2H), 2.41 (td, J = 6.3, 4.3 Hz, 2H), 2.17–2.09 (m, 1H), 2.05 (m, 2H), 1.95 (m, 1H) ppm

{6-[6-(3,4-dihydro-2H-pyran-6-yl)-1,2,4,5-tetrazin-3-yl]-3,4-dihydro-2H-pyran-2-yl}methyl N-(29-{[2-(2,5-dioxo-2,5-dihydro-1H-pyrrol-1-yl)ethyl]carbamoyl}-3,6,9,12,15,18,21,24,27-nonaoxanonacosan-1-yl)carbamate (9)

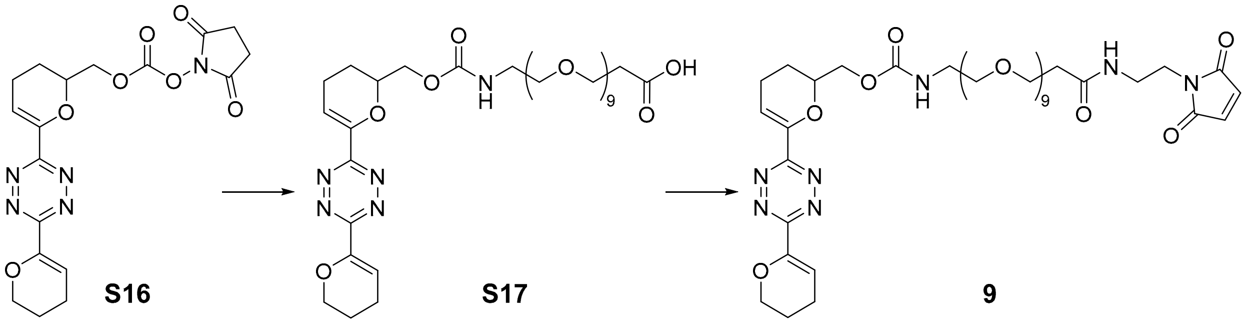

To a solution of compound **S16** (16.7 mg, 0.0312 mmol) in MeCN (0.5 mL) was added a solution of 1-amino-3,6,9,12,15,18,21,24,27-nonaoxatriacontan-30-oic acid (29 mg, 0.0598 mmol) in MeCN (0.5 mL), followed by DIPEA (12 mg, 0.0936 mmol). The pink solution was stirred at 20°C for 30 min, and subsequently formic acid (16 mg) and water (2 mL) were added. The product was purified by reversed-phase column chromatography (C_{18}, water/MeCN) and lyophilization to yield compound **S17** as a pink solid (18 mg, 73%). To a solution of compound **S17** (5.3 mg, 0.0067 mmol) in DMF (0.5 mL) was added DIPEA (4.3 mg, 0.033 mmol), N-(2-aminoethyl)maleimide TFA salt (2.1 mg, 0.0081 mmol), and PyBOP (5.3 mg, 0.010 mmol). The pink solution was stirred at 20°C for 30 min, and subsequently formic acid (10 mg) and water (1 mL) were added. The product was purified by reversed-phase column chromatography (C_{18}, water/MeCN). The affluent was concentrated in vacuo to remove MeCN, and the aqueous solution was extracted with CH_2Cl_2 (10 mL). The organic layer was dried over Na_2SO_4, filtered, and concentrated in vacuo to yield compound **9** as a pink oil (4.0 mg, 66%). Analysis **S17**: ESI-MS: m/z Calc. for $C_{35}H_{57}N_5O_{15}$ 787.39 Da; Obs. $[M + H]^+$ 788.42 Da. ^1H NMR (400 MHz, $CDCl_3$): δ 6.71 (m, 2H), 5.57 (t, J = 5.3 Hz, 1H), 4.43–4.31 (m, 5H), 3.77 (t, J = 6.1 Hz, 2H), 3.71 (s, 2H), 3.65 (m, 32H), 3.56 (t, J = 5.1 Hz, 2H), 3.38 (q, J = 5.4 Hz, 2H), 2.60 (t, J = 6.0 Hz, 2H), 2.48–2.34 (m, 4H), 2.04 (m, 2H), 1.86 (m, 1H) ppm. Analysis **9**: ESI-MS: m/z Calc. for $C_{41}H_{63}N_7O_{16}$ 909.43 Da; Obs. $[M + H]^+$ 910.50 Da. ^1H NMR (400 MHz, $CDCl_3$): δ 6.84 (br.s, 1H), 6.71 (m, 3H), 5.44 (t, J = 5.4 Hz, 1H), 4.43–4.31 (m, 4H), 3.8–3.5 (m, 40H), 3.56 (t, J = 5.1 Hz, 2H), 3.45 (m, 2H), 3.38 (q, J = 5.2 Hz, 2H), 2.43 (m, 4H), 2.04 (m, 2H), 1.88 (m, 1H) ppm

Supplementary Figures

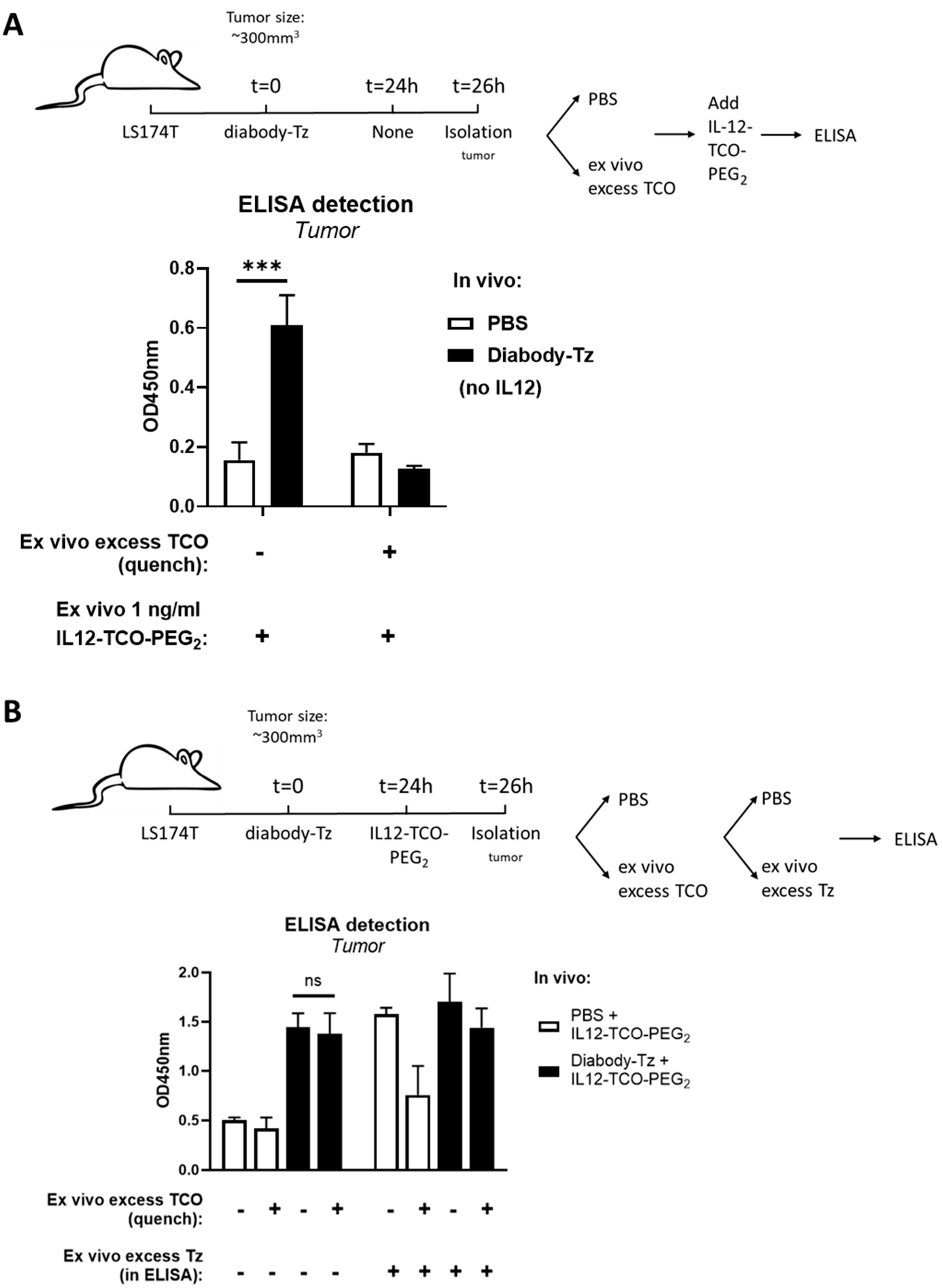

Figure S1. A) Control experiment demonstrating that Tz is still active during sample workup. ELISA analysis of tumor homogenate when pre-targeted diabody-Tz was quenched directly following isolation. To one-half of the samples (indicated with + below graph) a 100 eq molar excess of TCO **3** in protease inhibitor solution was added to deactivate all diabody-Tz present.

Unmasking of ex vivo added IL12-TCO-PEG$_2$ (indicated with + below graph) was next used to confirm that Tz remains intact during sample work-up. B) Control experiment demonstrating that, despite active Tz being present, the unmasking of IL12 constructs in tumors occurs in vivo and not during sample workup. Diabody-Tz was pretargeted to the tumor (black bars), and next, IL12-TCO-PEG$_2$ was administered. Directly after isolation, Tz activity was quenched by an excess of free TCO 3 (indicated with + below graph). Additionally, before samples were transferred to the ELISA, the remaining IL12-TCO-PEG was unmasked upon adding excess small-molecule tetrazine trigger **8** (indicated with + below graph). Data confirm that IL12 unmasking occurred in the tumor and was essentially complete in vivo. Data represent mean OD450nm values with SEM, (n = 4). Significance: ns indicates $p > 0.05$; *** indicates $p \leq 0.001$.

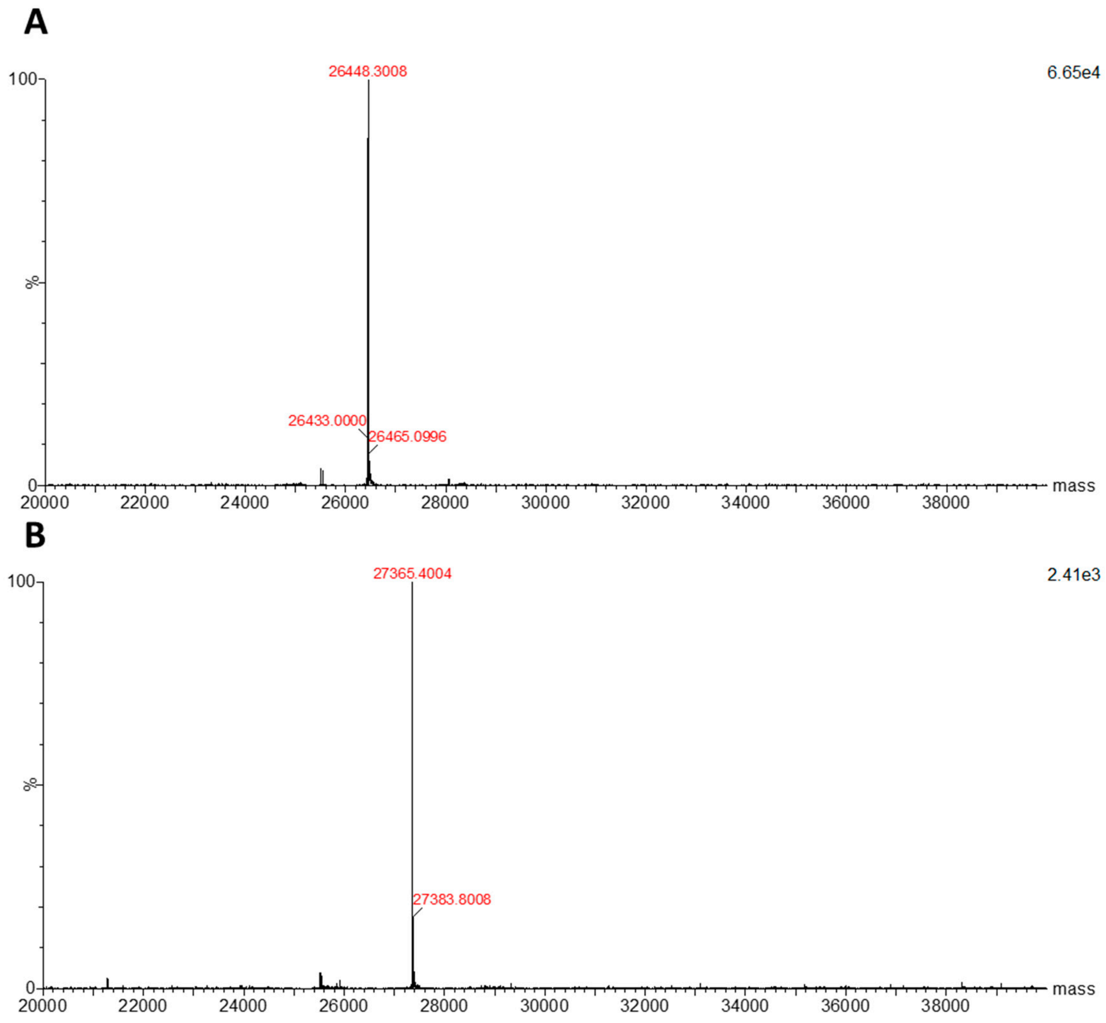

Figure S2. HPLC-QTOF-MS analysis of protein conjugates. A) MS spectrum after deconvolution for TCO-functionalized diabody **5**, showing mass of the diabody monomer with DAR of 2. B) MS spectrum after deconvolution for Tz-functionalized diabody **12**, showing mass of the diabody monomer with DAR of 2.

Spectra

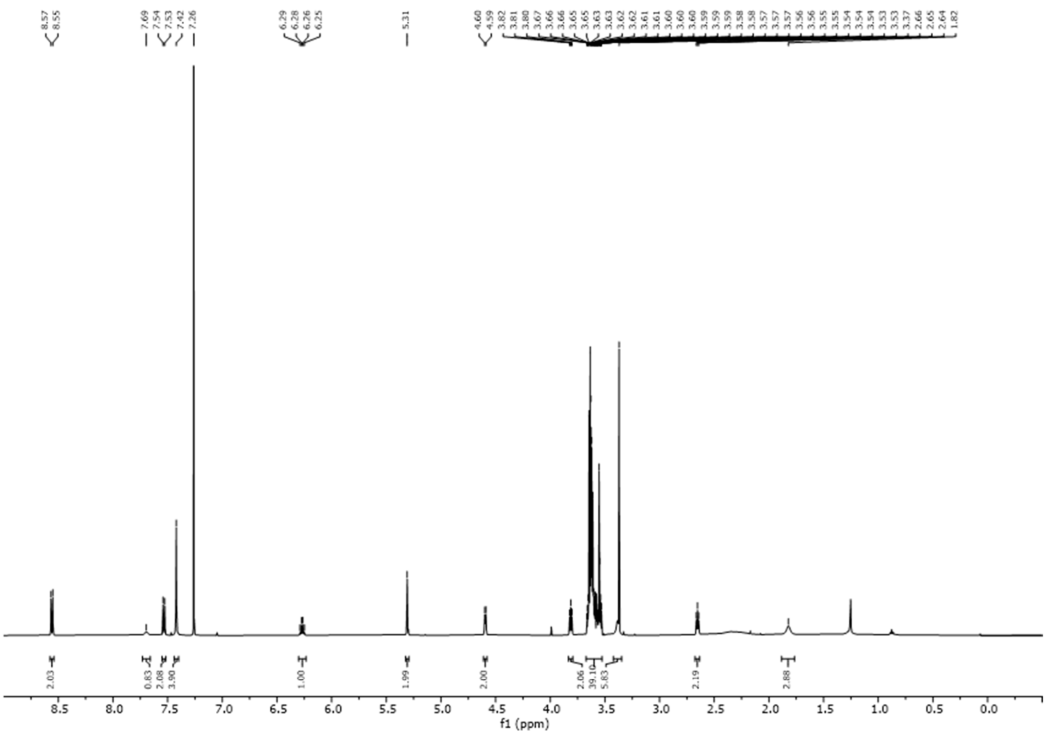

^{1}H-NMR spectrum ($CDCl_3$) of compound **1**

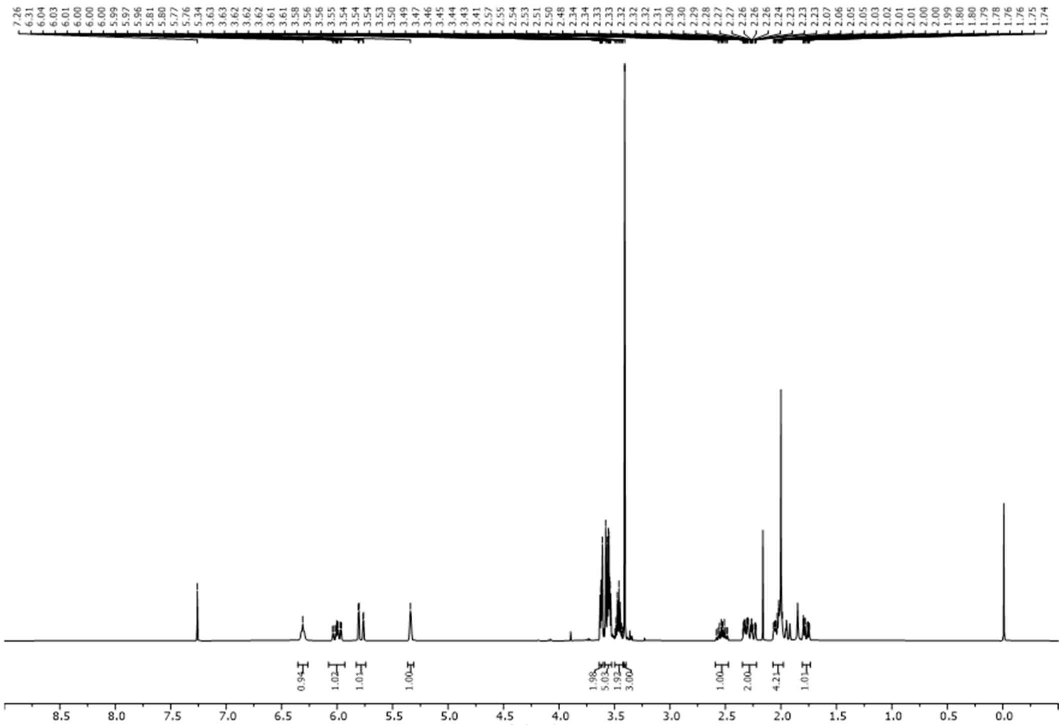

^{1}H-NMR spectrum ($CDCl_3$) of compound **6**

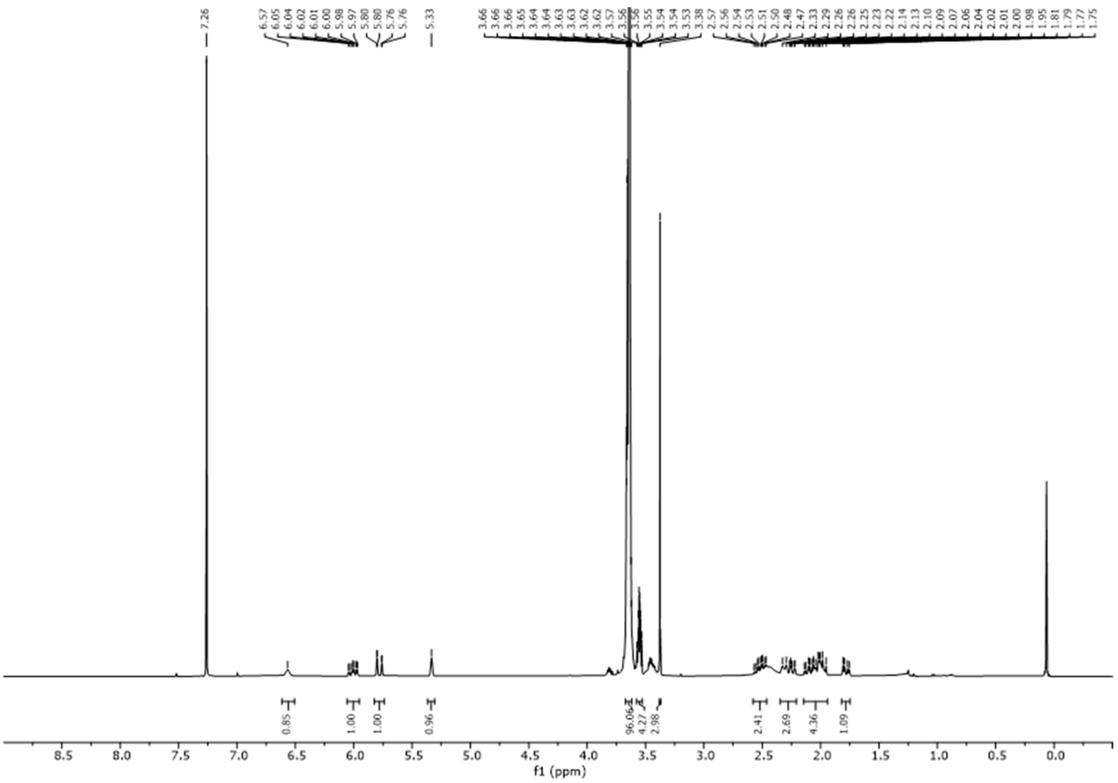

^{1}H-NMR spectrum ($CDCl_3$) of compound **7**

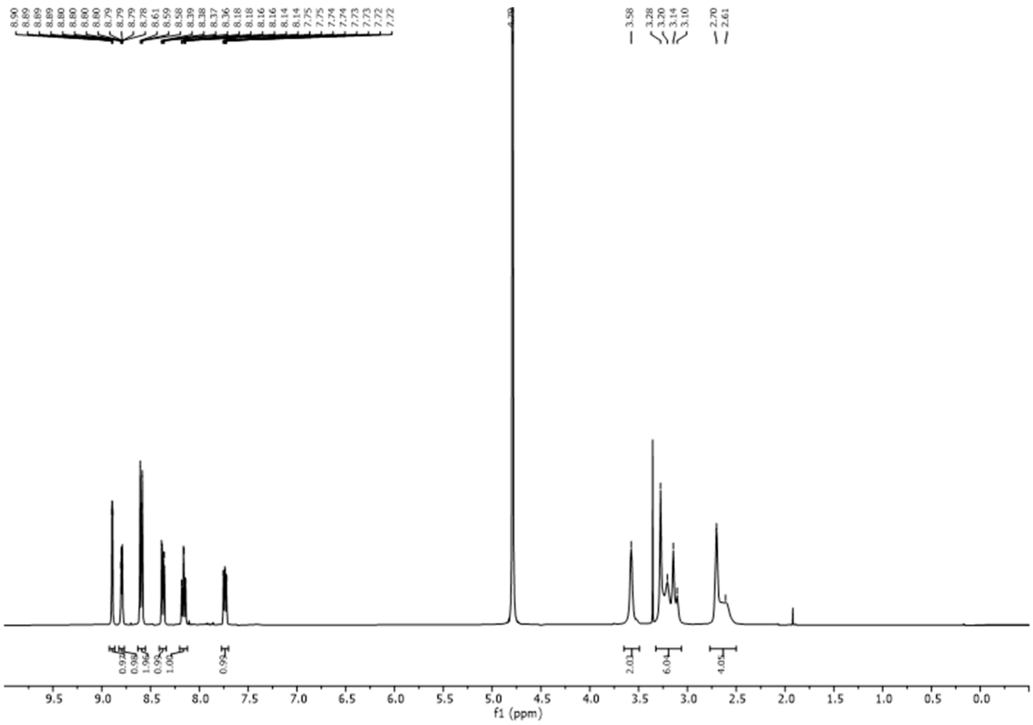

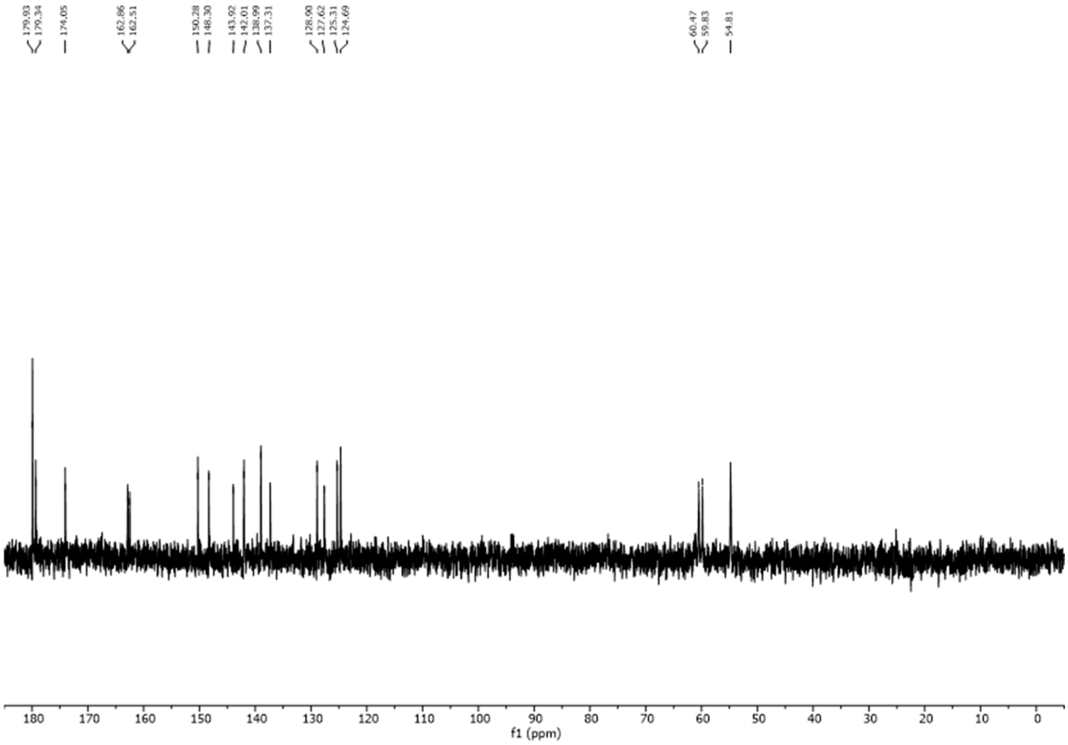

^{1}H- and ^{13}C-NMR spectra (D_2O) of compound **8**

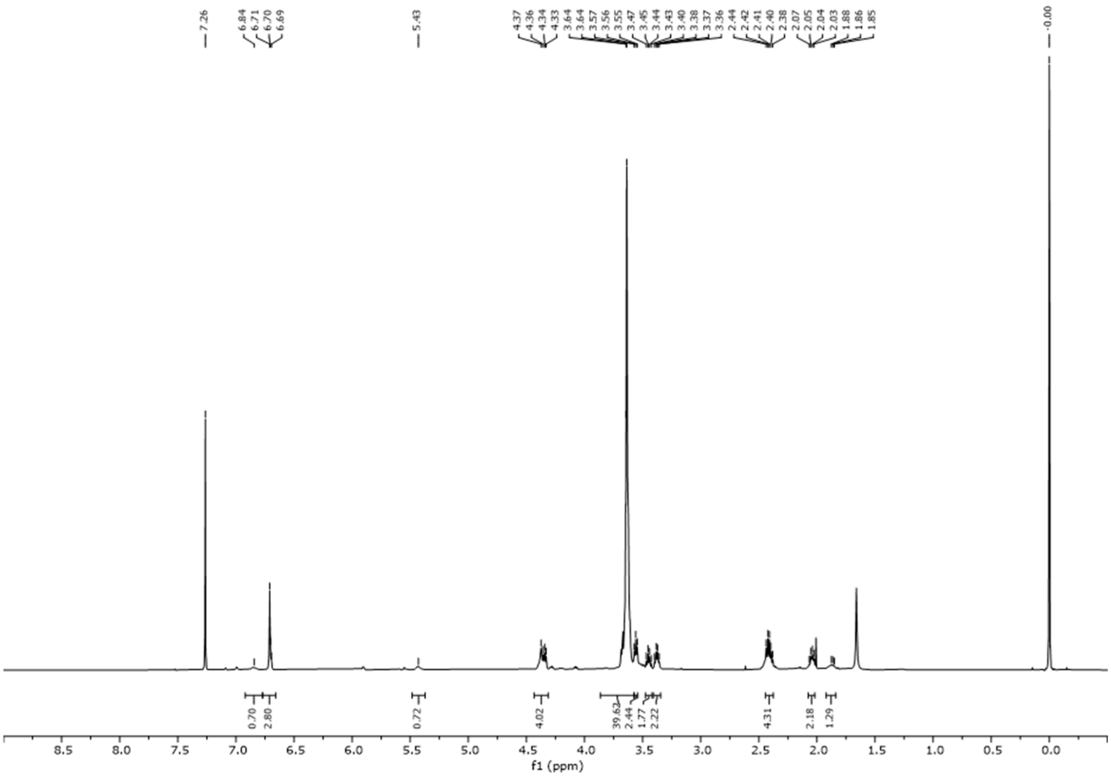

^{1}H-NMR spectrum ($CDCl_3$) of compound **9**

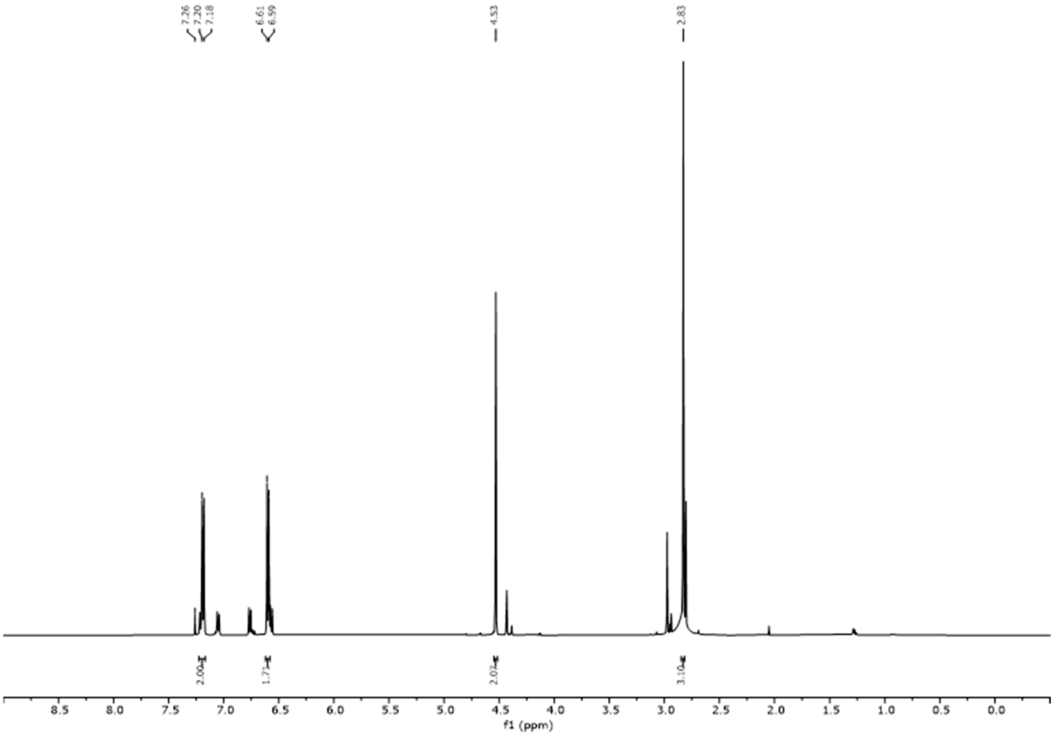

^{1}H-NMR spectrum ($CDCl_3$) of compound **S1**

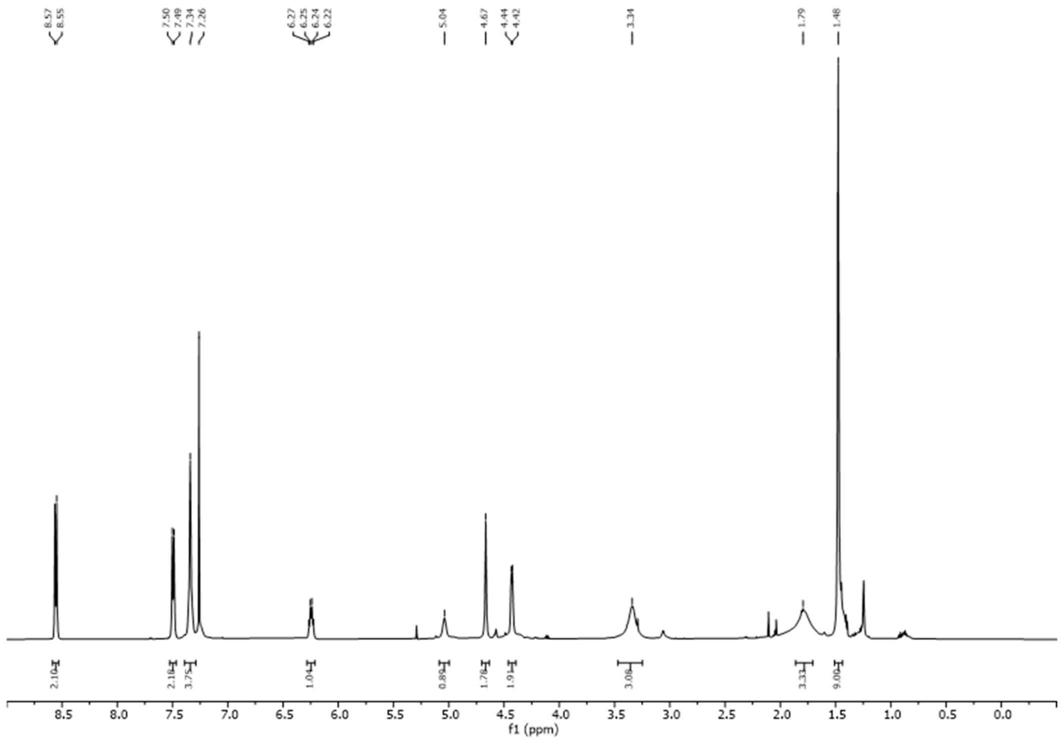

^{1}H-NMR spectrum ($CDCl_3$) of compound **S3**

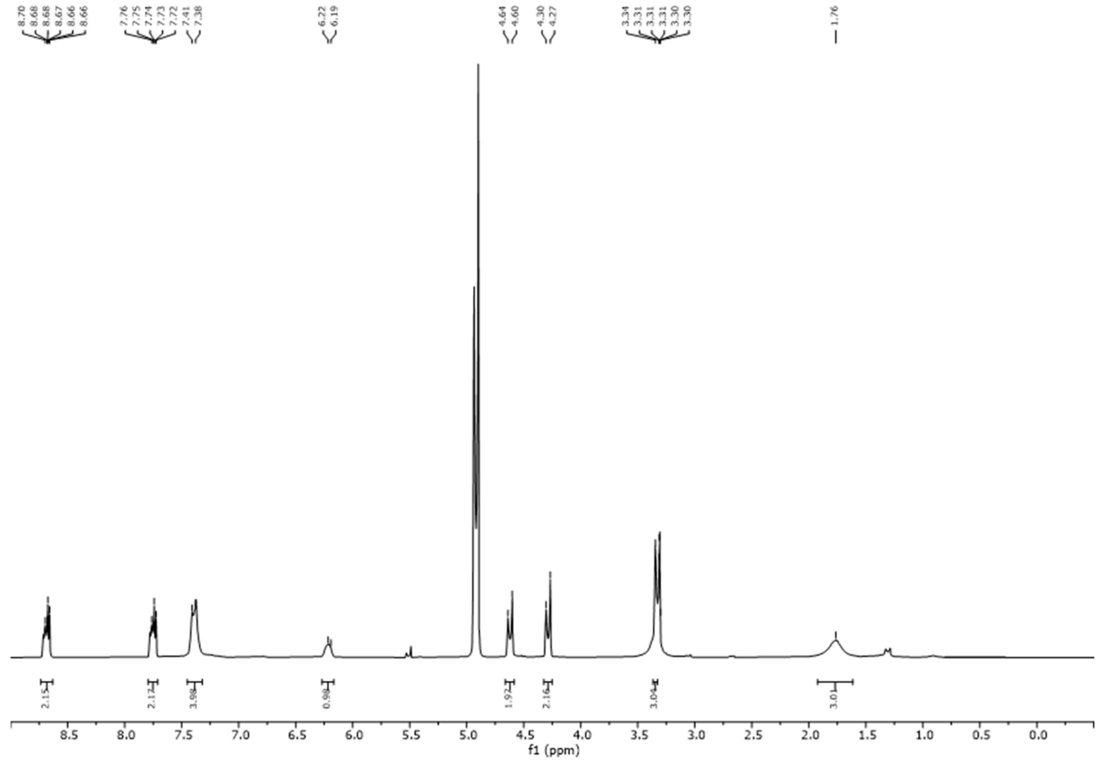

^{1}H-NMR spectrum (CD_3OD) of compound **S4**

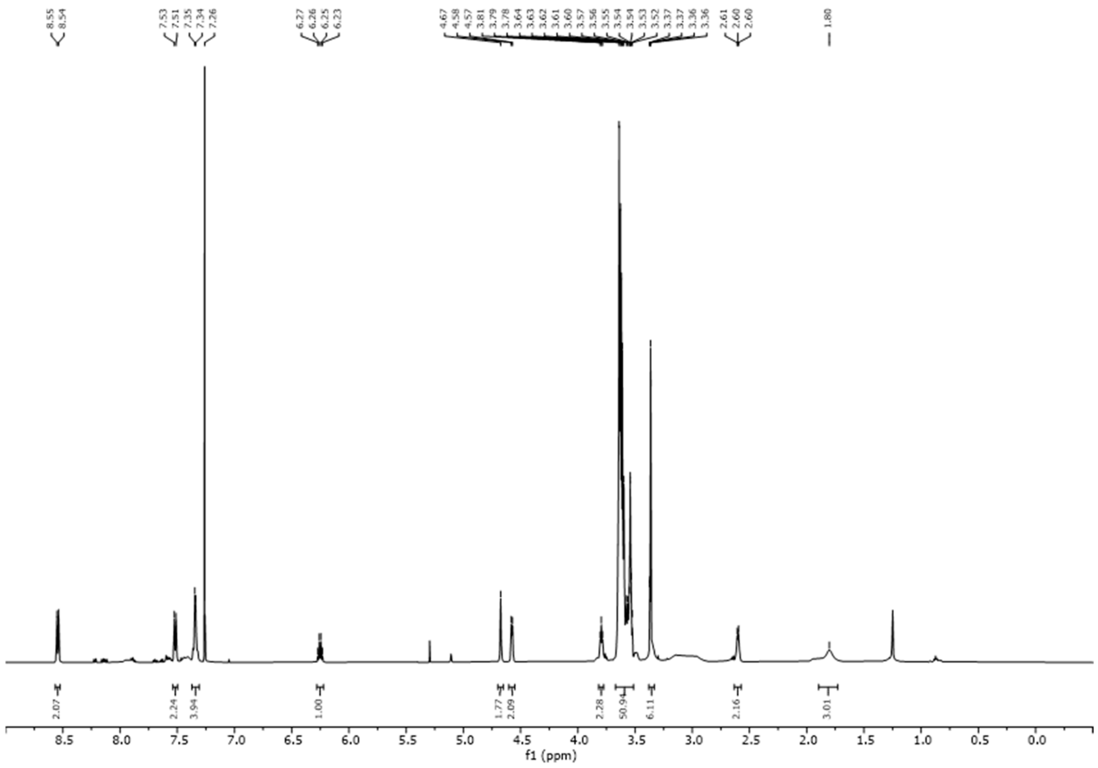

^{1}H-NMR spectrum ($CDCl_3$) of compound **S5**

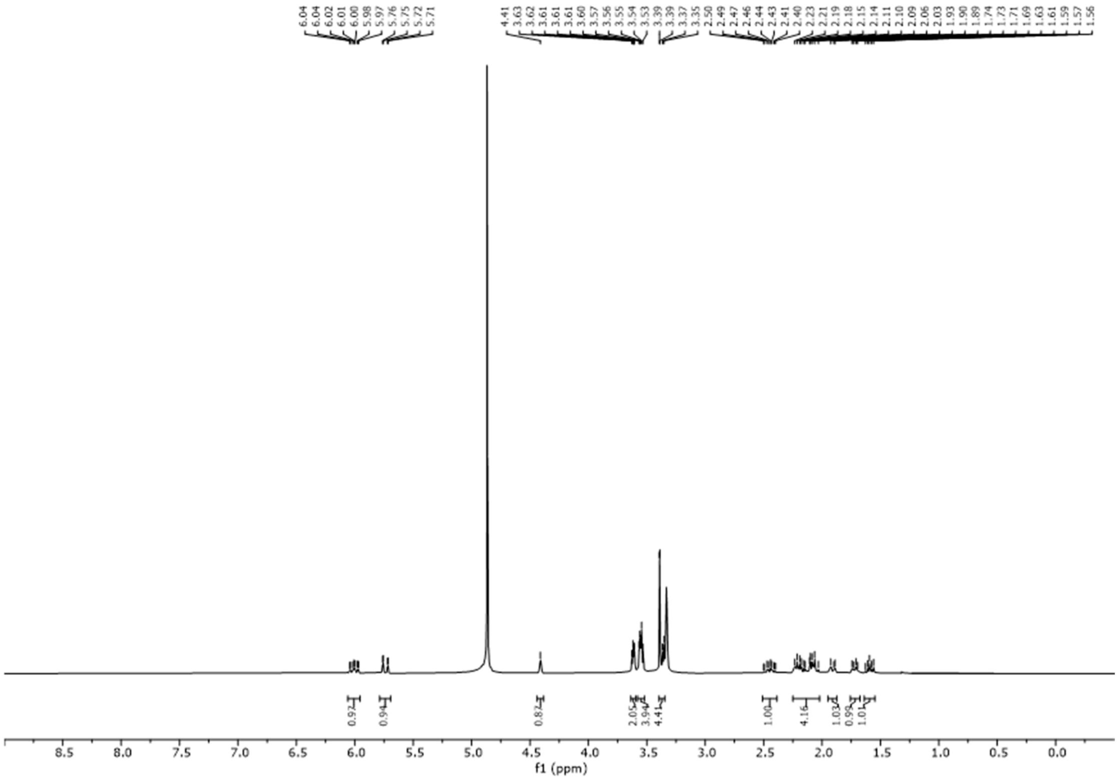

^{1}H-NMR spectrum (CD_3OD) of compound **S7**

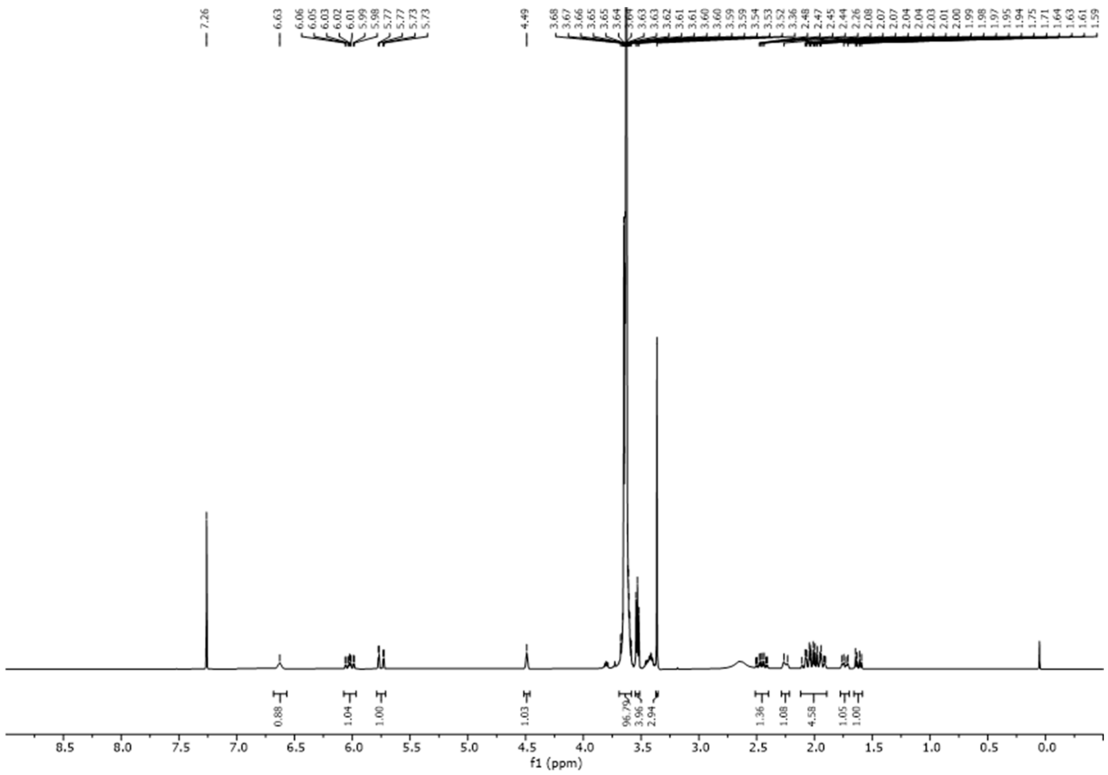

^1H-NMR spectrum ($CDCl_3$) of compound **S8**

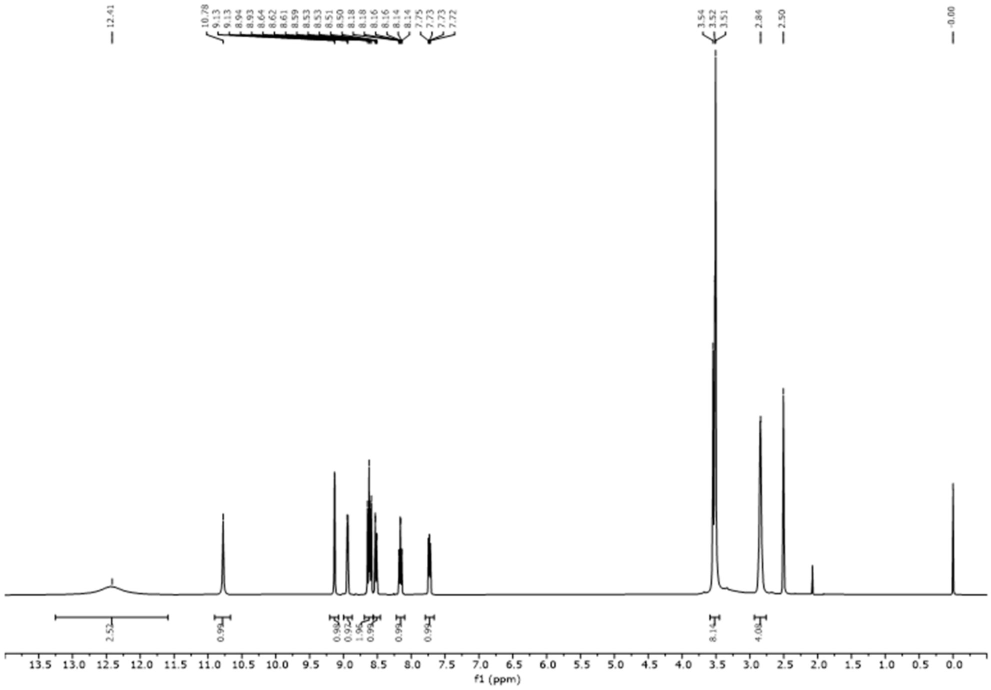

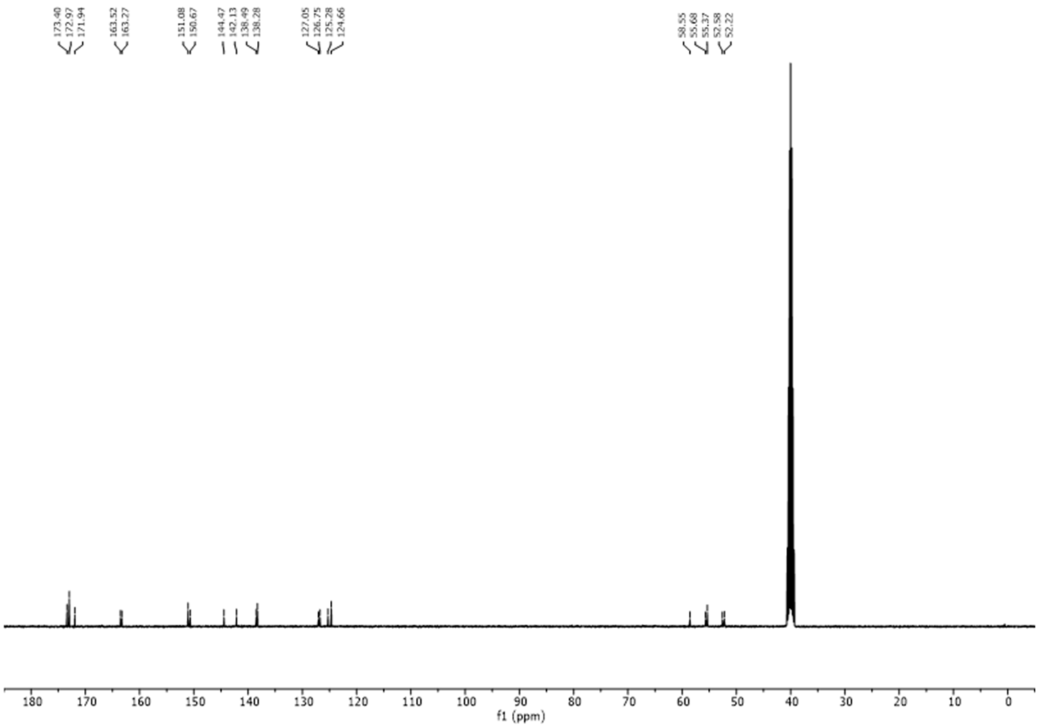

^{1}H- and ^{13}C-NMR spectra (DMSO-d_6) of compound **S11**

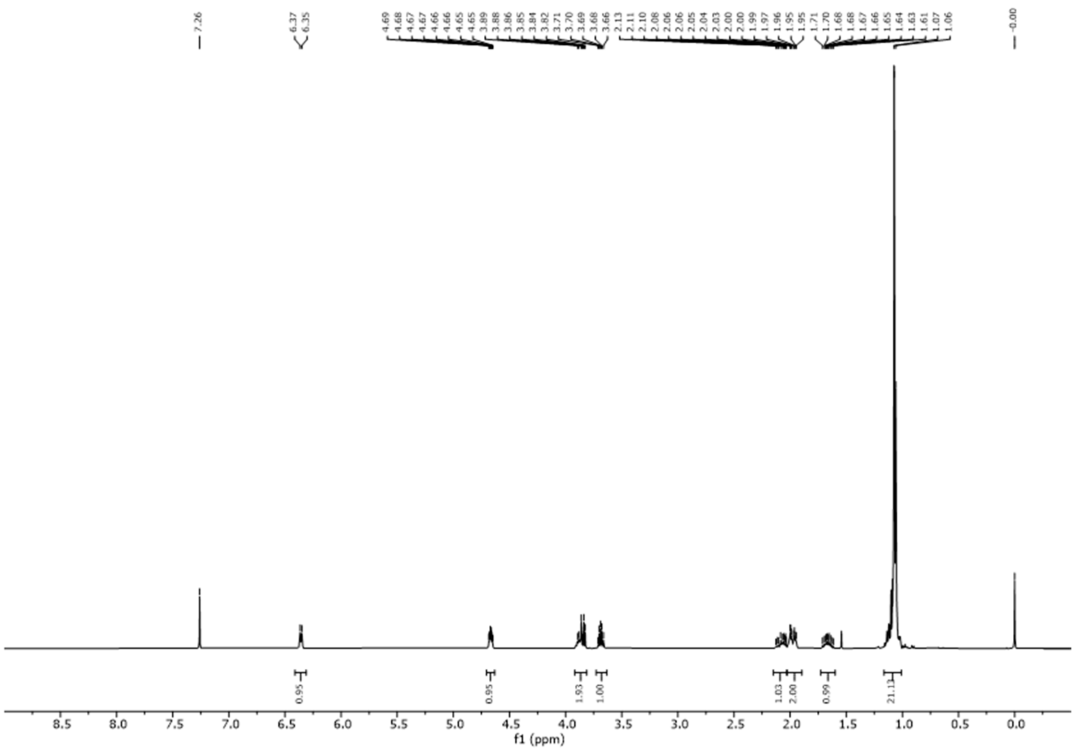

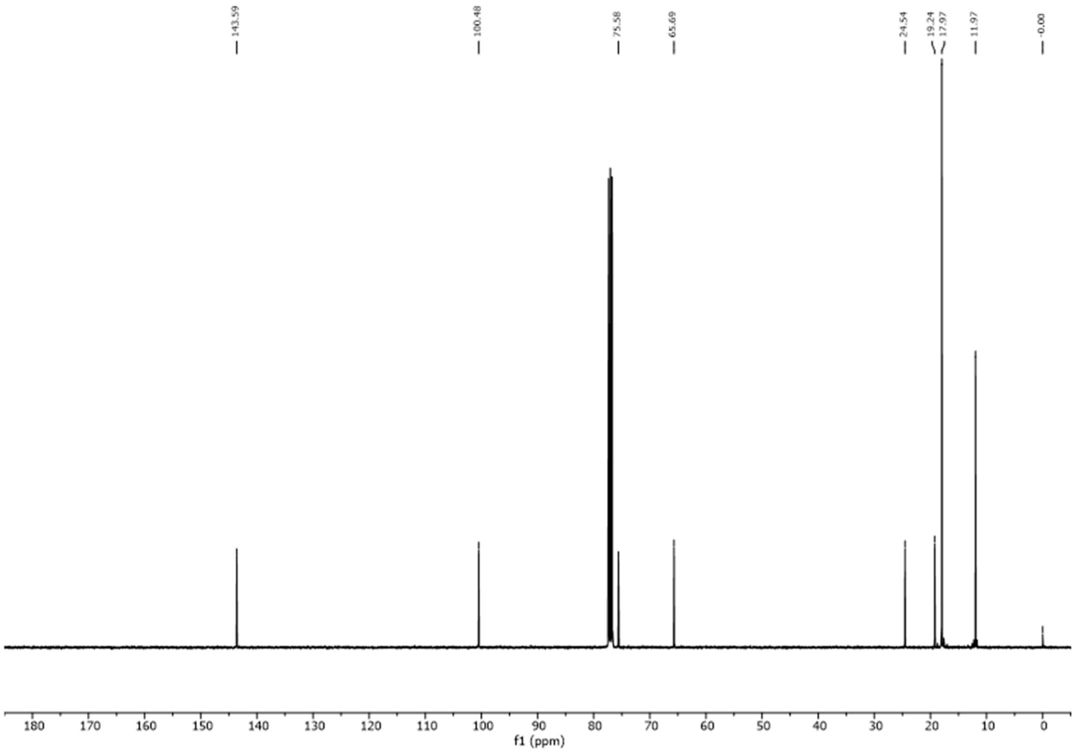

^1H- and ^{13}C-NMR spectra ($CDCl_3$) of compound **S12**

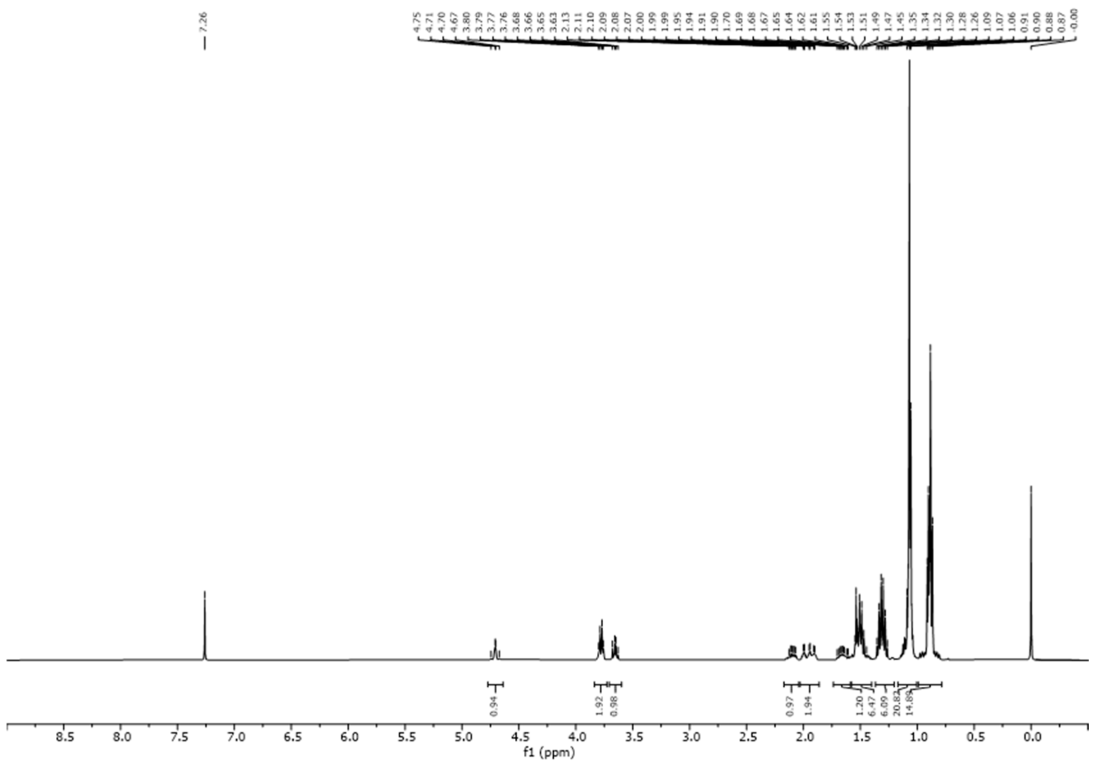

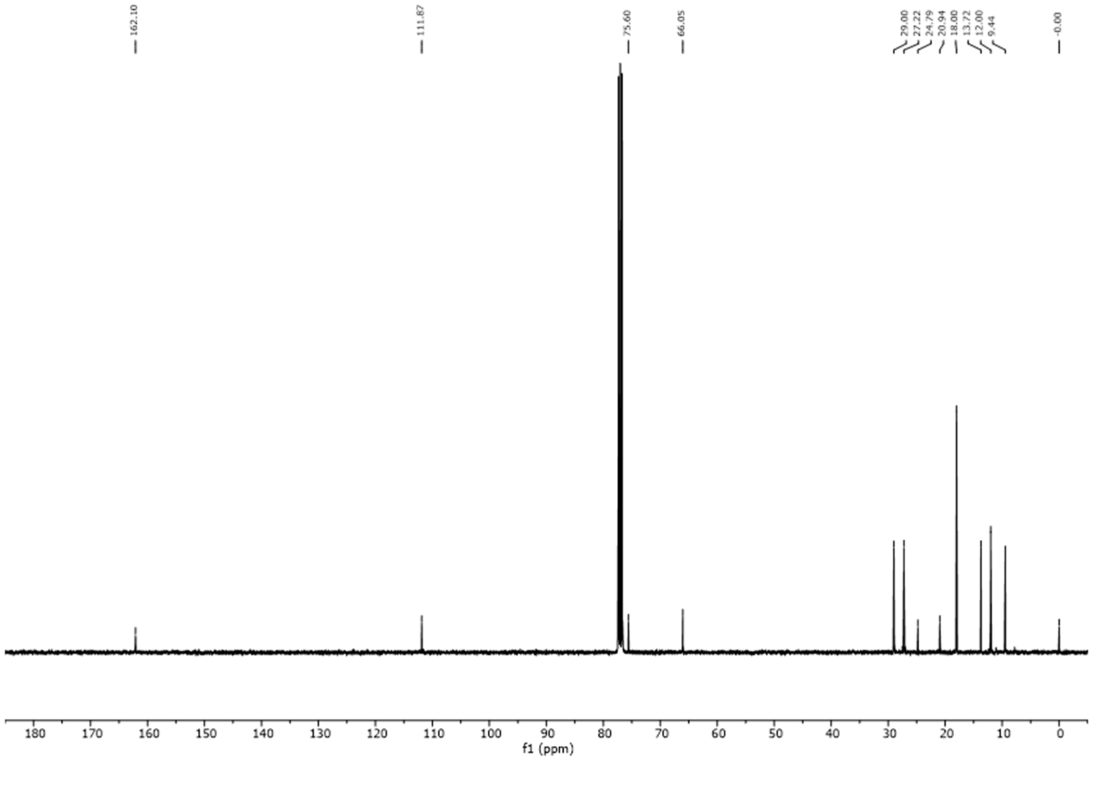

^1H- and ^{13}C-NMR spectra ($CDCl_3$) of compound **S13**

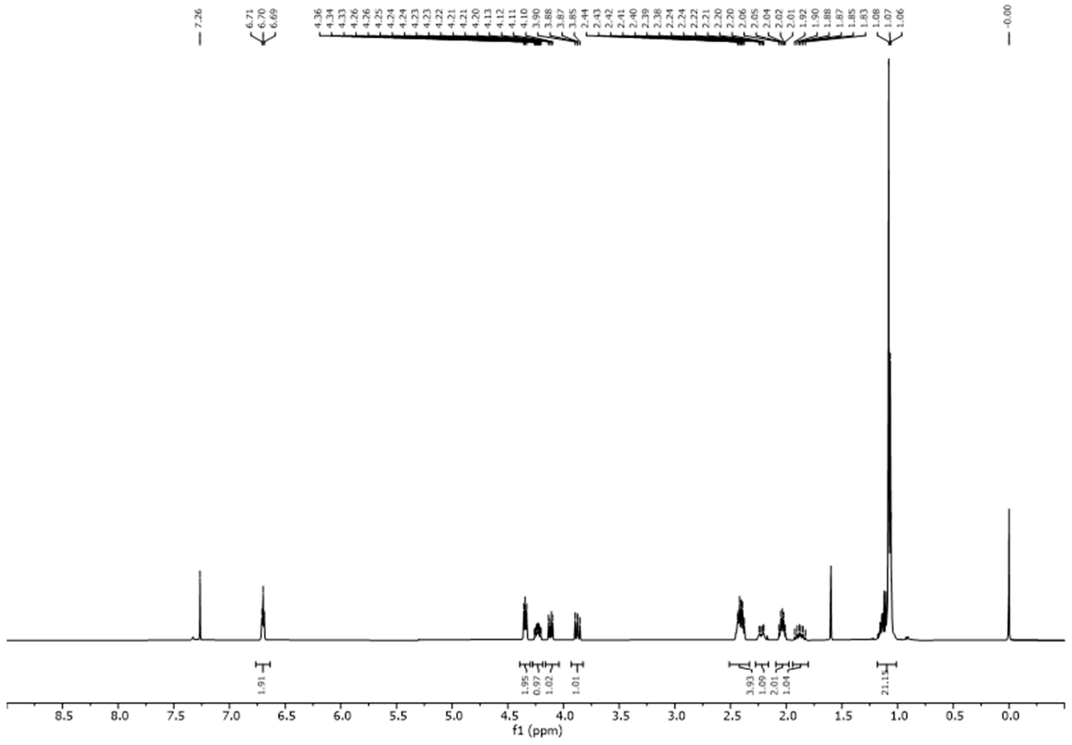

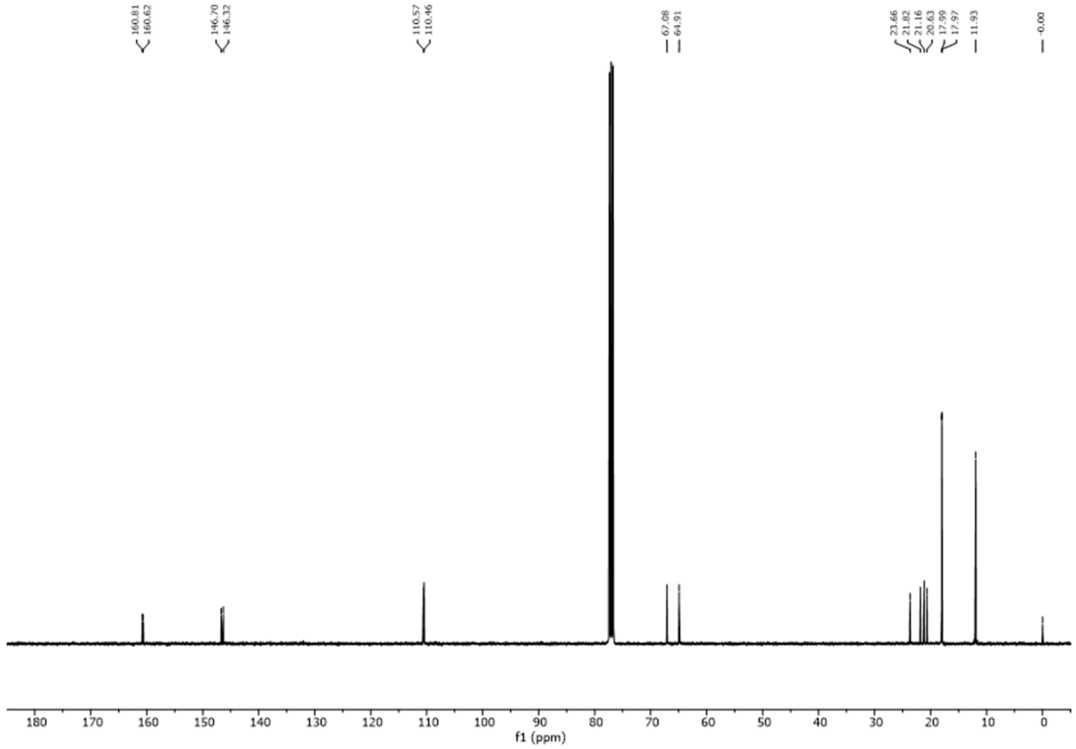

^{1}H- and ^{13}C-NMR spectra ($CDCl_3$) of compound **S14**

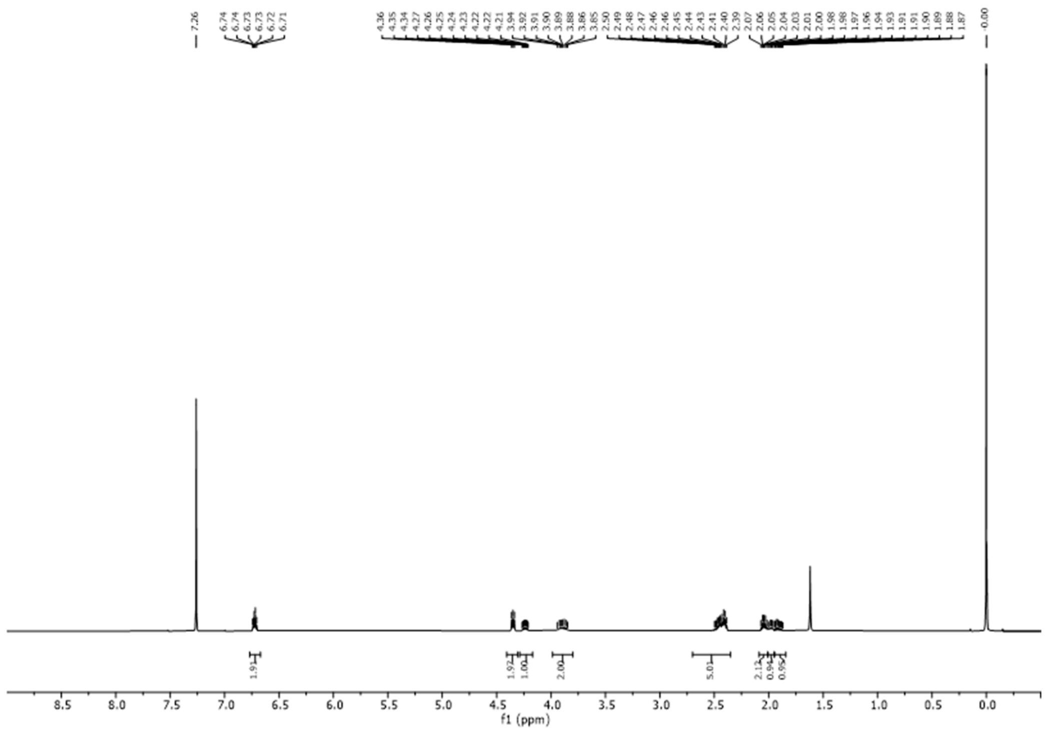

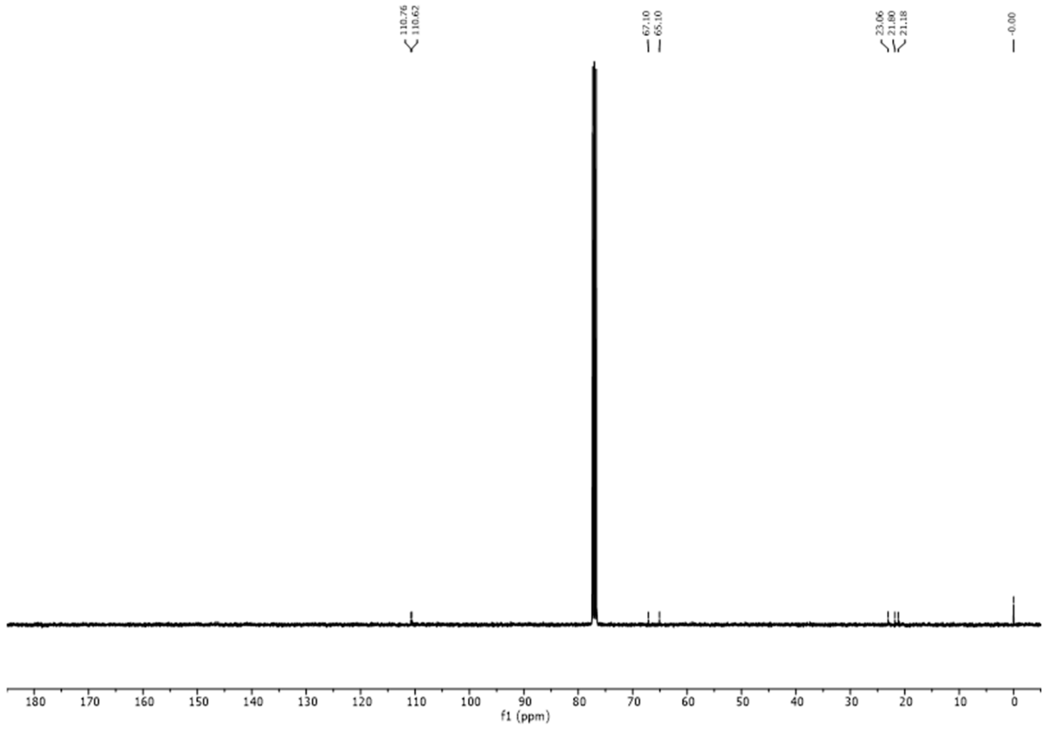

^{1}H- and ^{13}C-NMR spectra ($CDCl_3$) of compound **S15**

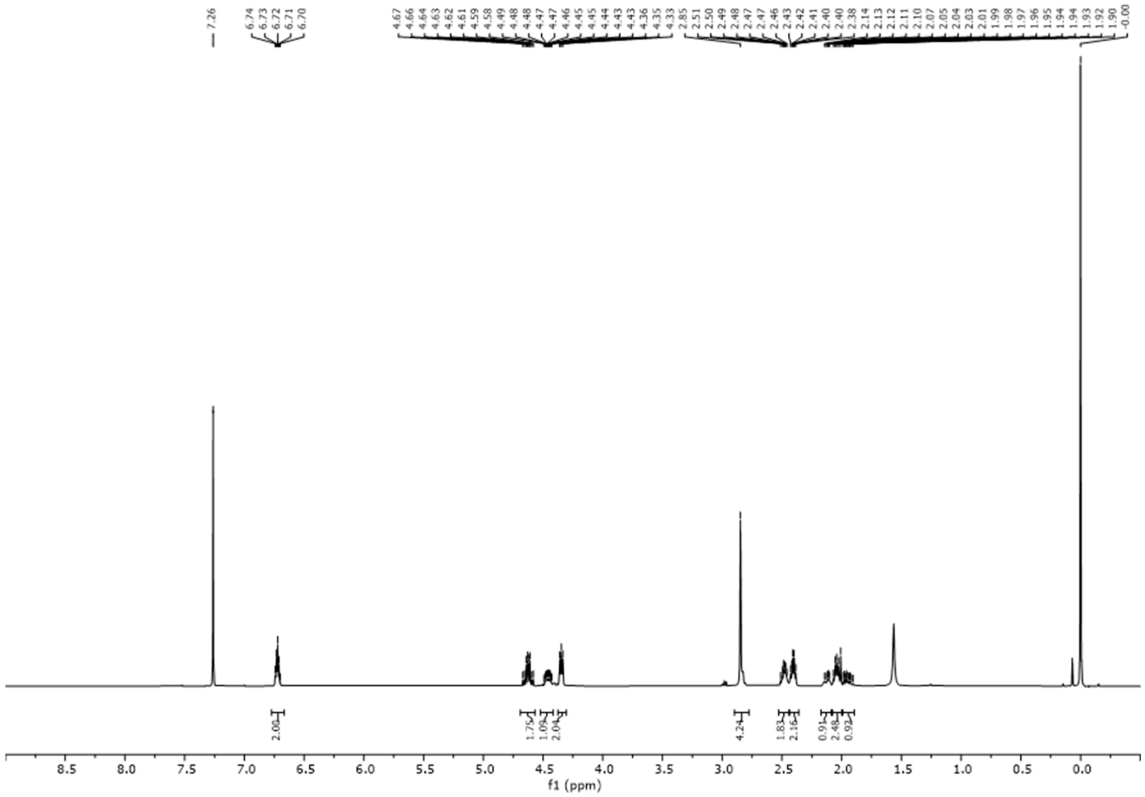

^{1}H-NMR spectrum ($CDCl_3$) of compound **S16**

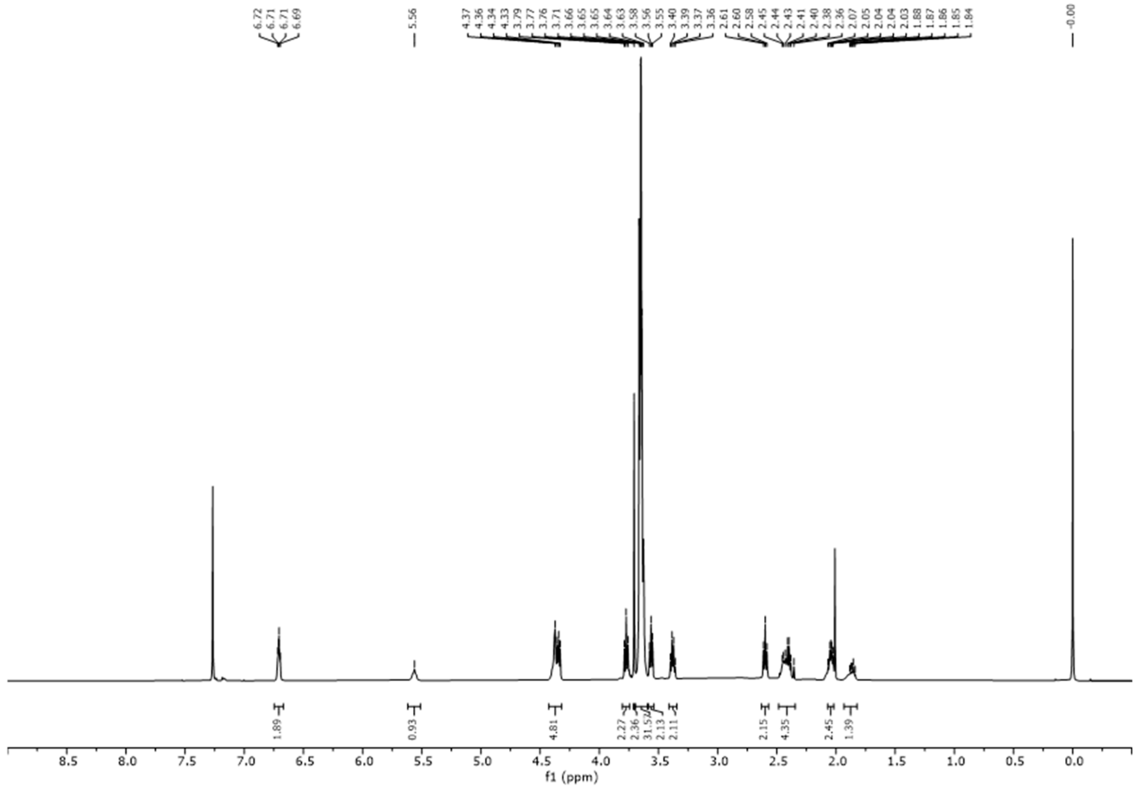

^{1}H-NMR spectrum ($CDCl_3$) of compound **S17**

References

27. van Onzen, A.; Versteegen, R.M.; Hoeben, F.J.M.; Filot, I.A.W.; Rossin, R.; Zhu, T.; Wu, J.; Hudson, P.J.; Janssen, H.M.; Ten Hoeve, W.; et al. Bioorthogonal Tetrazine Carbamate Cleavage by Highly Reactive trans-Cyclooctene. *J. Am. Chem. Soc.* **2020**, *142*, 10955–10963.
28. Rossin, R.; Versteegen, R.M.; Wu, J.; Khasanov, A.; Wessels, H.J.; Steenbergen, E.J.; Ten Hoeve, W.; Janssen, H.M.; van Onzen, A.; Hudson, P.J.; et al. Chemically triggered drug release from an antibody-drug conjugate leads to potent antitumour activity in mice. *Nat. Commun.* **2018**, *9*, 1484.
43. Rossin, R.; van Duijnhoven, S.M.; Läppchen, T.; van den Bosch, S.M.; Robillard, M.S. Trans-cyclooctene tag with improved properties for tumor pretargeting with the diels-alder reaction. *Mol Pharm.* **2014,** *11,* 3090–3096.
44. Blackman, M.L.; Royzen, M.; Fox J.M.; Tetrazine ligation: fast bioconjugation based on inverse-electron-demand Diels-Alder reactivity. *J Am Chem Soc.* **2008**, *130*, 13518–13519.
45 Rossin, R.; Verkerk, P.R.; van den Bosch, S.M.; Vulders, R.C.; Verel, I.; Lub, J.; Robillard, M.S. In vivo chemistry for pretargeted tumor imaging in live mice. *Angew Chem Int Ed Engl.* **2010**, *49*, 3375–3378.
